# Alterations in pituitary adenylate cyclase-activating polypeptide in major depressive disorder, bipolar disorder, and comorbid depression in Alzheimer's disease in the human hypothalamus and prefrontal cortex

**DOI:** 10.1017/S0033291723001265

**Published:** 2023-12

**Authors:** Zala Slabe, Rawien A. Balesar, Ronald W. H. Verwer, Joop J. Van Heerikhuize, Gwyneth A. Pechler, Maja Zorović, Witte J.G. Hoogendijk, Dick F. Swaab

**Affiliations:** 1 Netherlands Institute for Neuroscience, an Institute of the Royal Netherlands Academy of Arts and Sciences, Meibergdreef 47, 1105 BA Amsterdam, The Netherlands; 2 University of Ljubljana, Faculty of Medicine, Institute of Pharmacology and Experimental Toxicology, Korytkova 2, 1000 Ljubljana, Slovenia; 3 University of Ljubljana, Faculty of Medicine, Institute of Pathophysiology, Zaloška 4, 1000 Ljubljana, Slovenia; 4 Erasmus University Medical Centre, Department of Psychiatry, Doctor Molewaterplein 40, 3015 GD Rotterdam, The Netherlands

**Keywords:** PACAP, paraventricular nucleus, prefrontal cortex, major depressive disorder, bipolar disorder, depression in Alzheimer disease

## Abstract

**Background:**

Pituitary Adenylate Cyclase-Activating Polypeptide (PACAP) is involved in the stress response and may play a key role in mood disorders, but no information is available on PACAP for the human brain in relation to mood disorders.

**Methods:**

PACAP-peptide levels were determined in a major stress-response site, the hypothalamic paraventricular nucleus (PVN), of people with major depressive disorder (MDD), bipolar disorder (BD) and of a unique cohort of Alzheimer's disease (AD) patients with and without depression, all with matched controls. The expression of PACAP-(Adcyap1mRNA) and PACAP-receptors was determined in the MDD and BD patients by qPCR in presumed target sites of PACAP in stress-related disorders, the dorsolateral prefrontal cortex (DLPFC) and anterior cingulate cortex (ACC).

**Results:**

PACAP cell bodies and/or fibres were localised throughout the hypothalamus with differences between immunocytochemistry and *in situ* hybridisation. In the controls, PACAP-immunoreactivity-(ir) in the PVN was higher in women than in men. PVN-PACAP-ir was higher in male BD compared to the matched male controls. In all AD patients, the PVN-PACAP-ir was lower compared to the controls, but higher in AD depressed patients compared to those without depression. There was a significant positive correlation between the Cornell depression score and PVN-PACAP-ir in all AD patients combined. In the ACC and DLPFC, alterations in mRNA expression of PACAP and its receptors were associated with mood disorders in a differential way depending on the type of mood disorder, suicide, and psychotic features.

**Conclusion:**

The results support the possibility that PACAP plays a role in mood disorder pathophysiology.

## Introduction

Pituitary Adenylate Cyclase-Activating Polypeptide (PACAP) is a neuropeptide that is presumed to play a role in disorders that are characterised by enhanced activity of the neuroendocrine stress systems, such as in major depressive disorder (MDD), bipolar disorder (BD), post-traumatic stress disorder and schizophrenia (Katayama, Hattori, Yamada, Matsuzaki, & Tohyama, 2009). The hypothalamic paraventricular nucleus (PVN) is one of the major hubs in the stress response systems (Lucassen et al., 2014), in the pathogenesis of depression (Bao, Ruhé, Gao, & Swaab, 2012) and in suicide (Berardelli et al., 2020). Morphological and functional studies in rodents indicate that PACAP activates corticotropin-releasing hormone (CRH) neurons in the PVN. CRH neurons are the motor of the hypothalamic–pituitary–adrenal (HPA) axis (Mustafa, 2013). PACAP-containing terminals are present on CRH-containing perikarya and dendrites in the rat PVN (Hammack & May, 2015). Moreover, approximately 50% of the rat parvocellular PACAP-containing neurons simultaneously contain CRH (Hannibal, Mikkelsen, Fahrenkrug, & Larsen, 1995).

Our working hypothesis was that in stress-related conditions such as mood disorders and suicide, PACAP produced in the hypothalamic PVN, which is a main source of PACAP, was acting on the prefrontal cortex (PFC) by its receptors. The PFC is interconnected with the hypothalamus and involved in depression and suicide (Boucher, May, Braas, & Hammack, 2021; Ramikie & Ressler, 2016; Saper, 2000; Zhao et al., 2018).

PACAP acts via three G-protein coupled receptors: the PACAP type I receptor (PAC1), which exclusively binds PACAP, and two vasoactive intestinal peptide (VIP) receptors, VPAC1 and VPAC2 (Pinhasov et al., 2011; Rivnyak, Kiss, Tamas, Balogh, & Reglodi, 2018). In addition, PACAP has a fourth receptor, i.e. cyclic adenosine diphosphate ribose hydrolase (CD38) (Okamoto & Takasawa, 2002). PACAP has been demonstrated to interact synergistically with glutamate in the cerebral cortex to boost the ‘strength’ of glutamate-mediated signalling (Magistretti, Cardinaux, & Martin, 1998), while in the PFC glutamate is involved in depression and suicide (Zhao et al., 2015).

Mood disorders have emerged as one of the most significant chronic health issues at the present time (Dattani, Ritchie, & Roser, 2021). MDD is the leading cause of global disability (Friedrich, 2017). In addition, depression is a common comorbidity in Alzheimer's disease (AD), occurring in up to two-thirds of cases (Keller et al., 2017; Liu et al., 2018), while these patients are frequently resistant to conventional antidepressants (Cassano et al., 2019). A positive correlation between the Cornell score for depression severity in dementia (Alexopoulos, Abrams, Young, & Shamoian, 1988) and the number of CRH-expressing neurons in the PVN has been found earlier (Meynen, Unmehopa, Hofman, Swaab, & Hoogendijk, 2007).

There are some major questions in relation to PACAP and depression. Although hypothalamic CRH neurons are activated in MDD and BD (Belvederi Murri et al., 2016; Menke, 2019), and CRH production is increased in AD and mood disorders (Bao et al., 2012; Guo et al., 2022), no information is available about the possible involvement of PACAP in these disorders. Unexpectedly, previous studies (Chen, Du, & Chen, 2019; Han et al., 2014; Rat et al., 2011) found a downregulation of PACAP in AD in a number of brain regions.

Moreover, there are sex differences in the prevalence and symptoms of MDD and BD (Baskaran, Cha, Powell, Jalil, & McIntyre, 2014; Jogia, Dima, & Frangou, 2012; Labonté et al., 2017; Shors, Millon, Chang, Olson, & Alderman, 2017), while sex differences in the expression of PACAP and its receptors are also present in rodents (Mosca, Rousseau, Gulemetova, Kinkead, & Wilson, 2015; Ressler et al., 2011). Estradiol (E2) interacts and modulates PACAP signalling in stress-related brain regions that are associated with depression and anxiety, such as the bed nucleus of the stria terminalis (BNST), amygdala, hippocampus, PVN, and medial prefrontal cortex (King, Toufexis, & Hammack, 2017). In addition, it was found that PAC1 regulates the psychological and physiological responses to stress via an oestrogen response element embedded in the promotor region of the *Adcyap1r1* gene (Kirry et al., 2018; Ressler et al., 2011; Roman et al., 2014). However, no information is available on sex differences in PACAP in the human brain in relation to depression.

The present study starts with a description of the distribution of PACAP in the human hypothalamus using immunocytochemistry and *in situ* hybridisation. Subsequently alterations in PACAP-immunoreactivity (ir) are quantitatively determined in the hypothalamic PVN in MDD, BD, and depression in AD and compared to their respective controls. In addition, the mRNA changes in PACAP and its receptors were determined in the PFC, specifically the dorsolateral PFC (DLPFC) and anterior cingulate cortex (ACC) of MDD and BD patients. Special attention is also paid to the possible presence of sex differences and the relationship of PACAP and its receptor genes to features of suicide and psychosis in the PFC.

## Materials and methods

### Immunocytochemical and *in situ* hybridisation studies on the hypothalamus

Post-mortem human hypothalami were obtained from the Netherlands Brain Bank (NBB, Director Dr Inge Huitinga). The donors or their next of kin provided informed consent for a brain autopsy and the use of the tissue and clinical information for research purposes. The NBB research committee approved the protocols. Following the autopsy, the hypothalamic region was fixed in 4% formaldehyde (pH 7.4) for around 1 month at room temperature. Serial 6 *μ*m sections were made using a Leitz microtome (Leitz, Wetzlar, Germany).

Cross-reactivity of anti-PACAP antibody with VIP is a major point of concern, since the two precursor molecules and the two peptides have quite some amino-acid sequences in common. Interestingly, however, the fact sheet of the polyclonal rabbit-anti-PACAP antibody (anti-PACAP-38, T-4473, BMA, Peninsula Labs) stated that the cross-reactivity in a radioimmuno-assay of the PACAP-antibody used in the present study with VIP was 0%. We showed by Western, that despite 68% sequence homology between PACAP-38 and VIP, this anti-PACAP-38 antibody clearly reacted only with PACAP-38, and not with VIP peptide (see supplementary data SM6, Fig. SM1). In addition, we performed a solid phase adsorption to remove potential VIP cross-reacting antibodies from the anti-PACAP antibody following three solid phase adsorptions with VIP (Garcia-Falgueras, Ligtenberg, Kruijver, & Swaab, 2011; Van der Sluis, Pool, & Sluiter, 1987). BNST sections were subsequently stained using adsorbed antibody solution (SM7, Fig. SM2 panel B) and compared to the original, non-adsorbed anti-PACAP antiserum (SM7, Fig. SM2, panel A). The staining that remained after adsorption was quite similar to the original staining, indicating that potential VIP-cross reacting antibodies do not add significantly to the immunohistochemical signal produced by the anti-PACAP antibody. The control strip spotted with PACAP peptide and incubated with the same adsorbed PACAP-antibody solutions, exhibited largely preserved labelling (for details on the adsorption see SM7 and Fig. SM2). In addition, it should be noted that any possible cross-reactivity of anti-PACAP antibody with VIP would not interfere with our quantitative data in the PVN, since VIP fibres do not terminate in the human PVN but around this nucleus in the anteroventral hypothalamic area, the sub-PVN area and the dorsomedial hypothalamic nucleus, while VIP cell bodies were not observed in the PVN (Dai, Swaab, & Buijs, 1997). In other words, VIP seems largely absent in the human hypothalamic PVN.

#### Distribution of PACAP-ir and *in situ* hybridisation

**Figure 1.**
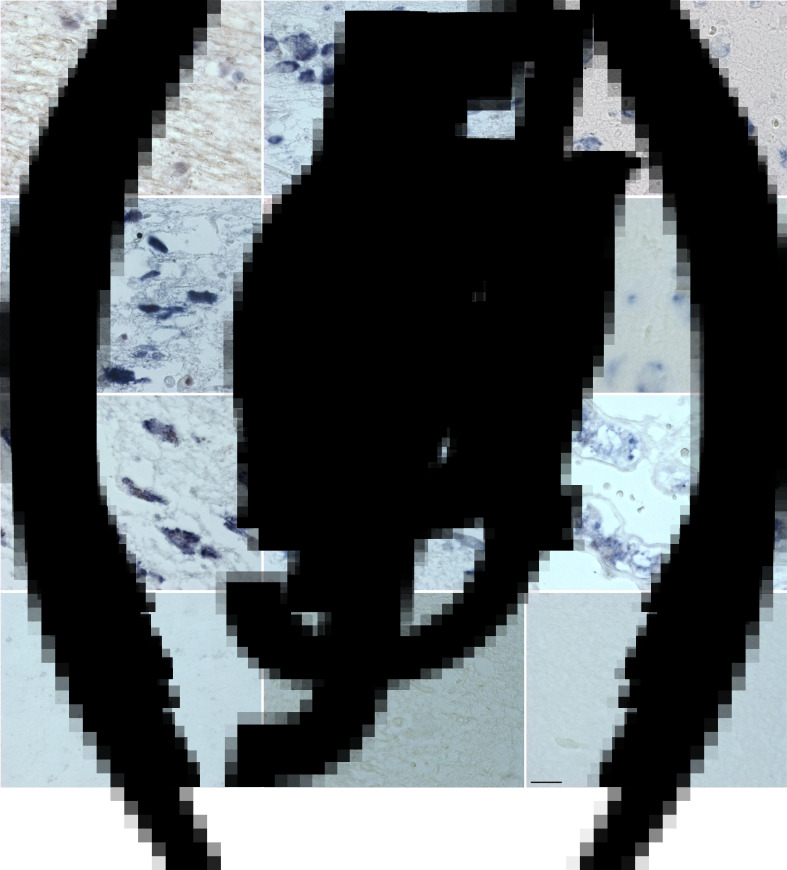
Distribution of PACAP mRNA in the human hypothalamus and adjacent areas. diagonal band of Broca (*a*), supraoptic nucleus (*b*), paraventricular nucleus (*c*), periventricular nucleus (*d*), infundibular nucleus (*e*), nucleus basalis of Meynert (*f*), tuberomammilary nucleus (*g*), lateral tuberal nucleus (*h*), pituitary, zona intermedia (*i*), central nucleus of the bed nucleus of stria terminalis (*j*), irrelevant probe (*k*) and scrambled probe (*l*). Calibration: 11.97 pixel/*μ*m. Bar: 1 mm.

**Figure 2.**
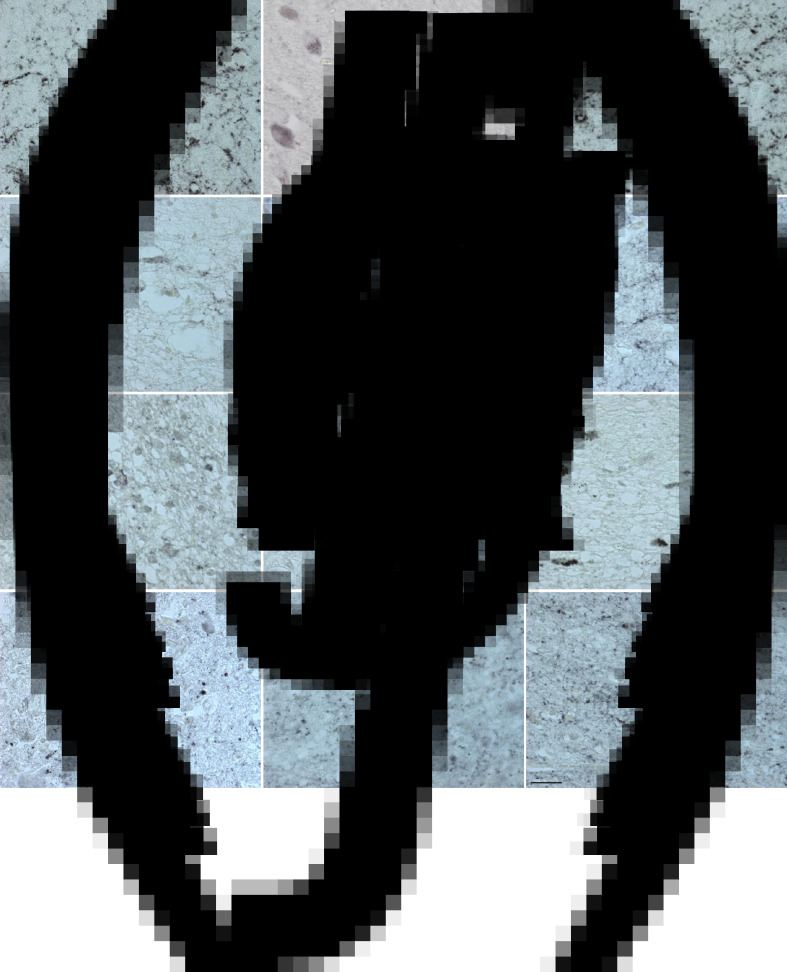
Distribution of PACAP immunoreactivity in the human hypothalamus and adjacent areas. central nucleus of the bed nucleus of stria terminalis (*a*), diagonal band of Broca (*b*), extended amygdala (*c*), lateral septum (*d*), nucleus basalis of Meynert (*e*), sexually dimorphic nucleus (*f*), suprachiasmatic nucleus (*g*), supraoptic nucleus (*h*), periventricular nucleus (*i*), paraventricular nucleus (*j*), ventromedial nucleus (*k*) and infundibular nucleus (*l*). Calibration: 0.77 pixel/*μ*m. Bar: 1 mm.

(Figures 1 and 2, online Supplementary Table S11). In order to describe the localisation of PACAP-ir in the different hypothalamic nuclei, 1:100 sections were stained in a male and a female control from rostral to caudal by anti-PACAP, thionine and specific immunocytochemical markers (see online Supplementary Table S11) to localise hypothalamic nuclei. All immunocytochemical stainings of PACAP were performed with the polyclonal rabbit-anti-PACAP antibody (T-4473, BMA, Peninsula Labs) at a dilution of 1:1500 as described in SM3.

In addition, the distribution of PACAP-mRNA (Adcyap1) was described in the human hypothalamus from rostral to caudal of 9 control women (age range from 32 to 85 years) and 8 men (age range from 44 to 92 years) using a locked Nucleic Acid probe for *in situ* hybridisation as described before (see SM5).

#### Mood Disorder patients and controls

A cohort of 17 patients (6 women; 11 men), with mood disorders was studied (see online Supplementary Table S1, 2), consisting of donors with MDD (*n* = 10) or BD (*n* = 7) and 17 controls (CTR *n* = 17) who were well matched (online Supplementary Table S1–2b) for sex, age, post-mortem delay (PMD), fixation time, clock time at death, month of death, brain weight and Braak stage for Alzheimer's neuropathology (Braak & Braak, 1991) (see online Supplementary Tables S1 and S2). Diagnosis of MDD or BD at any time during life was made by qualified psychiatrists according to the Diagnostic and Statistical Manual of Mental Disorders (DSM)-III-R/DSM-IV criteria (American Psychiatric Association, 1994). The absence of neuropathological changes, both in patients with mood disorders and in controls, was confirmed by systematic neuropathological investigation (van de Nes, Kamphorst, Ravid, & Swaab, 1998).

#### AD Patients with depression and without depression and controls

The AD patients, who had been studied at six-month intervals for depression within the framework of a prospective longitudinal study of depression in AD (online Supplementary Table S3 and for matching S3a and for patients SM1), were described in detail in our previous studies (Hoogendijk et al., 1999; Meynen et al., 2007). This cohort of 32 patients (10 men and 22 women) consisted of 14 patients who had confirmed AD but no depression, 9 who had confirmed AD and depression, and 9 were matched control subjects. The controls had no neurological or psychiatric disorder. The three groups of patients were well matched for pH, age (range from 64 to 96, mean±SEM: 78 ± 1.27), PMD and fixation time. Moreover, the two sub-groups of AD patients were matched for Braak stage (*p* = 0.67) (Braak & Braak, 1991). For details on matching see online Supplementary Table S3b). All patients were systematically neuropathologically investigated.

#### Quantification of PACAP-ir in the hypothalamic PVN

Immunocytochemical staining of PACAP took place on sections taken from the peak of the CRH staining area in the PVN in our previous study (Gao, Yang, Mack, & Chamberlin, 2022). Sections (3–5 per patient) were mounted on glass Superfrost TM Plus slides and put on hot plates for two nights at 38 °C.

The area of the PVN was manually outlined on every section on the basis of the borders of an adjacent thionine-stained section. To determine the PACAP-ir with the Zeiss Axiovert 200 M inverted microscope, black and white images were transformed into optical density (OD) images. The area of PACAP staining was determined by setting a threshold of 1.2 times the background OD. This prevented the inclusion of lipofuscin in the mask. The average OD multiplied by this area was used to give the integrated optical density (IOD). The IOD provides an estimate of the amount of PACAP staining in the outlined structure. Dividing the IOD by the outlined area of the PVN obtains the corrected density measure (cIOD).

### Quantitative real-time PCR (qPCR) study on the PFC

PFC brain samples were obtained from the Stanley Medical Research Institute (SMRI) (Bethesda, MD, USA, Director Dr Maree Webster). The next of kin provided consent for the material, via participating medical examiners. Diagnoses of MDD or BD were made according to the DSM IV (Bell, 1994). The SMRI provided RNA isolated from the post-mortem material of the ACC and DLPFC, all demographic information, and medical data including any lifetime use of psychotropic medication and a history of drug abuse (see online Supplementary Tables S4–S9). The depression collection consisted of 36 brain donors: 17 donors died of suicide. In addition, 12 MDD with psychotic features (MDD-P), 12 MDD with no psychotic features (MDD-NP), and 12 unaffected controls. From the MDD patients 17 died from suicide (MDD-S) and 7 from a natural death (MDD-N) (see online Supplementary Table S4a: for matching S4b). The ‘ARRAY’ collection consisted of 64 brain donors: 30 BD (17 non-suicidal patients 13 suicide completers) and 34 unaffected controls, From the BD patients 16 had psychotic features (BP-P), while 14 did not have psychotic features (BP-NP) (see online Supplementary Table S5a, for matching S5b). All patients were systematically neuropathologically investigated.

The (sub)-groups of each collection were matched for age, sex, brain weight, PMD and RIN value (see online Supplementary Table S4b–S9b). All the qPCR analyses were performed by investigators blind to the diagnosis. The cerebrospinal fluid (CSF) pH was lower in BD patients than in controls, but this is not considered to be a confounder but rather an endophenotype (See Discussion).

The mRNA expression of PACAP (Adcyap1) and its receptors, PAC1 (Adcyap1r1), VPAC1, VPAC2 and CD38, was determined by qPCR in the ACC and DLPFC of the controls and patients with mood disorder (for primers see online Supplementary Table S10).

For complementary DNA (cDNA) synthesis, an equal quantity of RNA (500 ng in a 10 *μ*l reaction mixture) for each sample was transcribed to cDNA using the QuantiTect Reverse Transcription Kit (Qiagen, Cat. No. 205313), after which the cDNA was used immediately or stored at −20 °C. qPCR was performed as we described before (see Zhao et al., 2018) (see SM 4).

### Statistics

#### For immunocytochemical staining

GraphPad Prism 8.1.1. (RRID:SCR_002798, GraphPad Software, 2019) was used for statistical analysis of the immunocytochemical data. Non-parametrical tests were used since the data were sometimes not normally distributed. The differences between two groups were evaluated by Mann–Whitney U test. Differences among more than two groups were first evaluated by means of the Kruskal–Wallis test and, if significant, were further evaluated by means of the Mann–Whitney U test between groups *p* < 0.05 was considered to be significant in all statistical tests.

#### For RT- qPCR analysis

S + software version 8.2 (TIBCO, 2010) was used for the statistical analysis of the qPCR results. Before processing the gene expression data, the values were 10log-transformed to enable simple reference gene correction and conventional statistical procedures. It may be noted that fold changes presented in Tables 1–3, online Supplementary Tables S12 and S13 are intended to illustrate the proportional difference between 2 groups and are not used in statistical tests. For interval data the Mann–Whitney test (2 samples) or the Kruskal–Wallis test with multiple comparisons (3 samples) was used (Conover, 1980). In multiple testing situations the Benjamini–Hochberg false discovery rate (FDR) adjustment (Benjamini & Hochberg, 1995) of *p* values was applied. All the tests were 2-sided. For analyses in multiple testing situations that showed at least one significant result after the FDR adjustment, we have provided 95% confidence intervals in the online Supplementary Figs (SR5–7). If in a set of multiple tests, none of the tests shows a significant result after FDR adjustment, it is impossible to construct confidence intervals because the corresponding critical level of significance is effectively zero. The confidence intervals for the Mann–Whitney test were calculated using all differences between the values of 2 groups (Conover, 1980). The central value of the confidence intervals for the Mann–Whitney test is the median of all these differences (called Hodges–Lehmann estimator). The adjusted interval lengths were obtained by inflating the confidence intervals according to the Benjamini–Hochberg procedure (95% false coverage rate (FCR) adjusted intervals). For the Kruskal–Wallis tests, the Benjamini–Hochberg corrected global *p* values were used to decide for which genes multiple comparisons were allowed. If multiple comparisons were allowed for only one gene of a set no further correction was needed (95% interval). However, when further testing of two or more genes in a set was allowed, subsequent Benjamini–Hochberg adjustments were applied to the multiple comparisons (95% FCR corrected intervals). Both *p* values and confidence intervals for multiple comparisons in the Kruskal–Wallis test were calculated using a rank version of Fisher's Least Significant Difference procedure (Conover, 1980). In this case, the test statistic is the difference between the mean ranks of two subdivisions and the x-axis of the confidence intervals is expressed in terms of ranks.

**Table 1.** PACAP related gene expression in the ACC and DLPFC in bipolar disorder (BD) and major depressive disorder (MDD) patients compared to matched controls

DLPFC				ACC		
	Fold change	*p* value	BH-adj	Fold change	*p* value	BH-adj
Bipolar disorder						
Target genes						
PAC1	−1.24	0.36		−1.02	0.67	
VPAC2	−1.46	0.51		−1.86	**0.001**	**0.004**
PACAP	−1.95	**0.01**	**0.034**	−1.42	**0.048**	0.08
VPAC1	−1.42	**0.003**	**0.013**	−1.49	**0.000048**	**0.0002**
CD38	−1.17	0.36		−1.34	0.38	
Major depressive disorder						
Target genes						
PAC1	2.0	0.075		1	0.57	
VPAC2	1.3	0.24		−1.25	0.4	
PACAP	1.98	**0.004**	**0.018**	1.16	0.37	
VPAC1	1.73	**0.048**	0.12	−1.1	0.61	
CD38	−1.06	0.42		1.36	0.87	

ACC, anterior cingulate cortex; BH-adj-P, *p* value following Benjamini–Hochberg's adjustment; CD38, cyclic adenosine diphosphate (ADP) ribose hydrolase; DLPFC, dorsolateral prefrontal cortex, – (before fold change), lower expression in BD; MDD, major depressive disorder; PAC1, PACAP type I receptor; VPAC1 and VPAC2; vasoactive intestinal peptide (VIP) receptors I and II.

**Table 2.** Relation to suicide

Fold change				Global-p	BHajd-p	BHajd-p		
	*CTR v*. *MDD-N*	*CTR v*. *MDD-S*	*MDD-N v*. *MDD-S*			*CTR v*. *MDD-N*	*CTR v*. *MDD-S*	*MDD-N v*. *MDD-S*
DLPFC target genes								
PAC1	2.40	1.81	−1.33	0.23	0.39			
VPAC2	1.35	1.31	−1.04	0.65	0.65			
PACAP	2.00	1.97	−1.02	**0.02**	0.1			
VPAC1	1.91	1.53	−1.25	0.14	0.36			
CD38	−1.23	−1.22	1.10	0.64	0.65			
ACC target genes								
PAC1	−1.55	−1.04	1.49	0.64	0.81			
VPAC2	−3.03	−1.34	2.26	0.81	0.81			
PACAP	−2.65	1.33	3.55	**0.006**	**0.03**	0.11*	0.03*	0.001*
VPAC1	−1.54	−1.04	1.49	0.66	0.81			
CD38	−1.47	1.09	1.60	0.34	0.81			

	*CTR v*. *BD-N*	*CTR v*. *BD-S*	*BD-N v*. *BD-S*			*CTR v*. *BD-N*	*CTR v*. *BD-S*	*BD-N v*. *BD-S*
DLPFC target genes								
PAC1	−1.19	−1.39	−1.17	0.39				
VPAC2	−1.30	−1.67	−1.28	0.78				
PACAP	−1.57	−2.10	−1.33	**0.05**	0.11			
VPAC1	−1.41	−1.32	1.07	**0.01**	**0.04**	**0.01**	**0.02**	0.84
CD38	−1.06	−1.47	−1.39	0.65				
ACC target genes								
PAC1	−2.22	−1.40	1.59	0.16				
VPAC2	−1.16	1.17	1.35	**0.003**	**0.008**	**0.001**	0.18	0.18
PACAP	−1.95	−1.05	1.85	**0.01**	**0.017**	**0.004**	0.92	**0.04**
VPAC1	−1.61	−1.53	1.05	**0.0002**	**0.001**	**0.0001**	**0.01**	0.37
CD38	−1.23	−1.34	−1.09	0.64				

PACAP related gene expression in the ACC and DLPFC in MDD suicide completers (MDD-S) and bipolar suicide completers (BD-S), MDD (MDD-N) and BD (BD-N) patients who died of natural causes, and their matched controls (CTR): ACC: anterior cingulate cortex; BD: bipolar disorder; BD-N: bipolar disorder patients that died of natural causes; BD-S: bipolar disorder patients that completed suicide; BHadj-P: *p* value of Benjamini–Hochberg's adjustment; Ctr: control; CD38: cyclic adenosine diphosphate (ADP) ribose hydrolase; DLPFC: dorsolateral prefrontal cortex; MDD: major depressive disorder; PAC1, PACAP type I receptor; VPAC1 and VPAC2; vasoactive intestinal peptide (VIP) receptors I and II.*: Correction not necessary.

**Table 3.** Relation to psychotic features

Fold change				Global-p	BHajd-p	BHajd-p		
	*CTR v*. *MDD-NP*	*CTR v*. *MDD-P*	*MDD-NP v*. *MDD-P*			*CTR v*. *MDD-NP*	*CTR v*. *MDD-P*	*MDD-NP v*. *MDD-P*
DLPFC target genes								
PAC1	2.08	1.99	−1.11	0.19	0.24			
VPAC2	−1.07	1.97	2.12	0.14	0.24			
PACAP	1.29	2.46	1.90	**0.0009**	**0.004**	0.17*	6.00×10^−5^*	0.003*
VPAC1	1.65	1.70	1.03	0.17	0.24			
CD38	−1.83	1.13	1.33	0.74	0.74			
ACC target genes								
PAC1	−1.27	−1.03	1.23	0.67	0.86			
VPAC2	−1.85	−1.72	1.08	0.45	0.86			
PACAP	1.08	−1.36	−1.46	0.59	0.86			
VPAC1	−1.27	−1.03	1.24	0.69	0.86			
CD38	−1.09	1.02	1.12	0.97	0.97			

	*CTR v*. *BD-NP*	*CTR v*. *BD-P*	*BD-NP v*. *BD-P*			*CTR v*. *BD-NP*	*CTR v*. *BD-P*	*BD-NP v*. *BD-P*
DLPFC target genes								
PAC1	−1.67	−1.04	1.61	0.09				
VPAC2	−1.12	−1.23	−1.10	0.87				
PACAP	−2.66	1.64	1.62	**0.03**	0.07			
VPAC1	−1.91	1.19	1.60	**0.0003**	**0.0013**	**0.00002**	0.36	**0.0012**
CD38	−2.50	1.02	2.56	0.16				
ACC target genes								
PAC1	−1.09	1.00	1.09	0.73				
VPAC2	−2.07	−1.58	1.31	**0.006**	**0.014**	**0.002**	**0.038**	0.25
PACAP	−1.50	−1.73	−1.15	0.10				
VPAC1	−2.04	−1.36	1.50	**0.0001**	**0.0004**	**0.00001**	**0.027**	**0.04**
CD38	−2.13	−1.09	1.95	0.27				

PACAP related gene expression in the ACC and DLPFC in MDD and BD patients with (MDD-P) and without (MDD-NP, BD-NP) psychotic features and their matched controls (CTR): ACC, anterior cingulate cortex; BD, bipolar disorder; BD-NP, bipolar disorder patients that died of natural causes; BD-S, bipolar disorder patients that completed suicide; BHadj-P, *p* value of Benjamini–Hochberg's adjustment; Ctr: control; CD38, cyclic adenosine diphosphate (ADP) ribose hydrolase; DLPFC, dorsolateral prefrontal cortex; MDD, major depressive disorder; PAC1, PACAP type I receptor; VPAC1 and VPAC2; vasoactive intestinal peptide (VIP) receptors I and II. *: Correction not necessary.

## Results

### Distribution of PACAP-ir and PACAP-mRNA in the human hypothalamus

(Figures 1 & 2 and online Supplementary Table S11a and Fig. 11b). Cells and fibres that contained PACAP-ir and cells that contained PACAP-mRNA appeared to be present throughout the hypothalamus and adjacent areas. There were, in some areas, clear differences between the two procedures. In the PVN there was a strong accumulation of densely packed PACAP-mRNA staining cell bodies, while PACAP-ir cell bodies were much fewer in number and only weakly staining. The central nucleus of bed nucleus of the BNST did not show any *in situ* mRNA signal, while it displayed the most intense staining of PACAP-ir nerve fibres often showing as baskets around negatively staining neurons. While PACAP-ir fibres and basket staining around cell bodies were present in the sexually dimorphic nucleus, medial preoptic area, suprachiasmatic nucleus, ventromedial nucleus, lateral septum and medial nucleus of the mammillary body, there were no PACAP mRNA containing cell bodies found in these nuclei. An accumulation of densely packed PACAP-mRNA positive cell bodies was detected in the tuberomamillary nucleus and some cell bodies were found by *in situ* hybridisation in the lateral tuberal nucleus, but there were no PACAP-ir expressing nerve fibres present in these nuclei. Although PACAP-ir cell bodies and fibres were present in the medial mammillary nucleus, there were no PACAP-mRNA containing cell bodies detected in this nucleus.

Similarities between the two approaches were present in the nuclei of diagonal band of Broca, where PACAP-ir cell bodies, baskets and fibres were present, as well as positive PACAP-mRNA cell bodies. Furthermore, PACAP-ir and PACAP-mRNA positive cell bodies were present in the nucleus basalis of Meynert, periventricular nucleus and PVN, while in the infundibular nucleus there were also fibres and baskets. Agreement between the immunocytochemistry and *in situ* hybridisation was also found in the lateral mammillary nucleus, where positive staining cell bodies were observed with both techniques.

### PACAP-ir Changes in the hypothalamic PVN in mood disorders

(see online Supplementary Figs SR1, SR2, and for matching Tables S1b, 2b). PACAP-ir in the PVN was significantly higher in the female controls, compared to the matched male controls (*p* = 0.0048, online Supplementary Fig. SR1).

PACAP-ir was significantly higher in the PVN of male BD patients compared to the matched male controls (*p* = 0.007, online Supplementary Fig. SR2). Furthermore, male BD patients had higher PVN-PACAP-ir levels than the MDD male group (*p* = 0.017, online Supplementary Fig. SR2).

### PACAP-ir Changes in the hypothalamic PVN in AD with or without depression

(online Supplementary Table S3a and S3b and Figs SR4 and SR5). Compared to controls the entire AD group had significantly lower PACAP-ir in the PVN (*p* = 0.0075). However, this difference turned out to be entirely due to the AD patients without depression, who had a highly significant lower PVN-PACAP level compared to the controls (*p* = 0.0012). AD patients with depression had a significantly higher PVN PACAP-ir than AD patients without depression (*p* = 0.0003). There was a significant positive correlation between the Cornell depression score and the expression of PACAP-ir in the PVN of the entire group of AD patients (Rho = 0.44, *p* = 0.034).

### mRNA Alterations of PACAP and its receptors in the DLPFC and ACC in MDD and BD

(Table 1 and online supplementary Figs SR5a–c). In the PFC the main mRNA changes in BD were a decreased mRNA expression of VPAC1 and VPAC2 in the ACC as compared to controls. In the DLPFC of BD patients there was a downregulation of PACAP and VPAC1 as compared to controls. In MDD, in the DLPFC the main change was an increased mRNA expression of PACAP as compared to controls.

#### Sex differences in mRNA alterations of PACAP and its receptors in the DLPFC and ACC in MDD and BD

No significant sex differences were found in the mRNA expression of PACAP, VPAC1, PAC1, VPAC2 or CD8 the ACC or DLPFC either in the controls or in the MDD or BD patients (see online Supplementary Tables S12–S13, for matching see Table S4b and S5b).

#### mRNA Alterations in PACAP and its receptors in the DLPFC and ACC in MDD and BD in relation to suicide

(Table 2 and online Supplementary Figs SR6–c). Changes in mRNA expression of PACAP and its receptors were present in relation to suicide (S) in both DLPFC and ACC areas. Especially in the ACC, PACAP-mRNA was upregulated in MDD-S compared to MDD-NS (NS = no-suicide, patients died from natural causes). PACAP-mRNA was also upregulated in BD-S compared to BD-NS.

#### mRNA Alterations in PACAP and its receptors in the DLPFC and ACC in MDD and BD in relation to psychotic features

(Table 3 and online Supplementary Figs SR7a–c). PACAP-related gene expressions were dysregulated in ACC and DLPFC in relation to psychotic features (P). In the DLPFC, PACAP-mRNA was upregulated in MDD-P in comparison to MDD-NP (NP = no psychotic features). In addition, VPAC1 was upregulated in the DLPFC in BD-P in comparison to BD-NP. In the ACC, VIPC1 and −2 were upregulated in BD-P in comparison to BD-NP.

#### Correlations of mRNA alterations in PACAP and its receptors in the DLPFC and ACC in MDD and BD with medication and pH

Antipsychotics were calculated as equivalents of fluphenazine doses (in milligrams) during the lifetime of a patient (see online Supplementary Table S14). There were mainly negative correlations observed between antipsychotics and mRNA expression of PACAP-related genes in MDD and BD patients.

Positive correlations between CSF pH and the mRNA expression of PACAP, VPAC1 and VPAC2 were found in the ACC of BD patients, and between CSF pH and PACAP and pH in the ACC of BD-P patients. In BD-S, there were negative correlations found between CSF pH and the mRNA expression and CD38 and PAC1 respectively. For all data see SM (online Supplementary Table S15).

## Discussion

### PACAP distribution in the human hypothalamus

The descriptive part of this study shows that both at the peptide and the mRNA the level, PACAP-containing neurons are present, while, in addition, PACAP-ir fibres are present throughout the human hypothalamus. The distribution of PACAP in the hypothalamus was generally in line with previous research on human tissue (Takahashi et al., 1994; Vigh et al., 1991). In our *in situ* hybridisation experiments, we found similar high numbers of PACAP-mRNA positive neurons in the PVN and in the supraoptic nucleus (SON) as these authors reported using immunocytochemistry, which reinforces the presence of PACAP staining neurons in these nuclei. Hashimoto et al. (1996) found a very strong *in situ* signal in the rat SON and a clear one in the PVN, similar to our results in human hypothalamus.

We examined in particular PACAP of MDD and BD patients in the PVN, since this nucleus is the motor of the stress response (Bao & Swaab, 2018). We found not only PACAP-ir fibres, but also PACAP-ir and PACAP-mRNA containing neurons in the PVN (see Figs 1 and 2). PACAP-ir fibres have been shown to innervate PVN neurons in rodents, while PACAP is co-localised with CRH and activates the stress-related CRH neurons (Hammack & May, 2015; Légrádi, Hannibal, & Lechan, 1998; Mustafa, 2013). Rodent research has shown that PACAP may play a role in the regulation of the hypothalamo–pituitary–adrenal (HPA) axis in response to psychogenic stress (Lehmann, Mustafa, Eiden, Herkenham, & Eiden, 2013) and in the regulation of the sympathetic nervous system (Hashimoto et al., 2011).

### Quantitative PACAP-ir changes in the hypothalamic PVN in mood disorders

We found that PACAP-ir was significantly elevated in the PVN of male BD patients compared to male controls. It should be noted that the number of female BD patient was too small to draw the conclusion of a sex difference here. There is indeed evidence from a mutant animal model that PACAP administration results in signs of mania, which is a central symptom of BD (Hashimoto et al., 2001). In contrast, we have not found any significant difference in PVN PACAP-ir in MDD patients, as proposed by others (Hashimoto et al., 2010; Pinhasov et al., 2011). Our study suggests that PACAP may be involved in particular symptoms of MDD such as suicide see mRNA changes in PACAP and receptor genes in BD and MDD in suicide) and psychotic features (see mRNA expression alterations in PACAP and its receptor genes in BD and MDD inrelation to psychotic features) rather than be related to MDD as such.

### Quantitative Alterations in PACAP-ir in the hypothalamic PVN in AD: relation to depression

It has been proposed that diminished PACAP in AD would have a diminished neurotrophic role that would contribute to this disorder, as indicated by lower levels of PACAP in the CSF in AD patients (Han et al., 2014). This possibility was, at first glance, in line with the diminished levels of PACAP-ir in the PVN of the AD patients that we observed. However, here we show that the diminished PACAP-ir was present only in AD patients that had no depression, and not in the AD patients with depression. The AD patients with depression had higher PACAP-ir in the PVN than AD patients without depression. It should be noted that both the depressed and non-depressed AD patients were in the end stage of the AD process, as indicated by their Braak scores (see online Supplementary Table S3). Therefore, the reported diminished PACAP–ir levels in AD seem to be related to the selected group of non-depressed AD patients, rather than to the AD process itself. A fascinating finding is the positive correlation we observed between the Cornell score for depression severity and the level of PVN PACAP-ir. Earlier our group found, in the same patients, a positive correlation between the Cornell score for depression severity in dementia and the number of CRH-expressing neurons in the PVN (Meynen et al., 2007). This suggests that PACAP may be involved in depression in AD by stimulation the central release of CRH, in a similar way as proposed for other mood disorders (Bao & Swaab, 2018; Holsboer & Ising, 2021).

### mRNA Alterations of PACAP and its receptors in the DLPFC and ACC in BD and MDD

The PFC is interconnected with the hypothalamus and is another major brain site that is involved in depression and suicide (Boucher et al., 2021; Dedovic, Duchesne, Andrews, Engert, & Pruessner, 2009; Gajewski, Turecki, & Robison, 2016; Price & Drevets, 2010; Ramikie & Ressler, 2016; Saper, 2000; Zhao et al., 2012, 2018, 2016).

Moreover, variations in PACAP receptors have been identified as a risk factor for psychiatric disorders, including mood disorders (Hayata-Takano et al., 2019). Our working hypothesis was that in stress related conditions such as mood disorders, PACAP from the hypothalamic PVN, which is a main source of PACAP production and a hub in the stress response, was acting on the PFC by PACAP receptors. The PFC is interconnected with the hypothalamus and involved in depression and suicide. However, PACAP-mRNA was also found in the PFC, and the levels showed changes in different directions in depression and suicide. PACAP-mRNA expression of in the prefrontal cortex has also been reported in rodents (Zhang et al., 2021), but its regulation in mood disorder and suicide was unexpected. In the ACC there was a downregulation of VPAC1 and VPAC2 in BD, and in the DLPFC we found a downregulation of PACAP and VPAC1 in BD DLPFC as compared to controls. In MDD, the main change was an increased mRNA expression of PACAP in the DLPFC. This shows that the human PFC has its own local PACAP system, with PACAP production and the presence of its receptors that have depression-related changes, as found for stress-related changes in rodents (Martelle et al., 2021).

#### mRNA Changes in PACAP and receptor genes in BD and MDD in suicide

We found an increased PACAP-mRNA expression in the ACC in both BD and MDD patients with accomplished suicide, as compared to mood disorder patients who died from natural causes. Interestingly, in an earlier study we found an increased mRNA expression of PACAP, VPAC1 and VPAC2 in the ACC of schizophrenia patients who completed suicide as compared to schizophrenic patients who died from natural causes (Slabe et al., 2023). This means that the increased mRNA expression of PACAP in relation to suicide is independent of the underlying neuropsychiatric disorder. The same holds for the transmitter activation of the ACC in suicide. We have previously observed increased expression of genes related to glutamatergic and GABAergic synaptic transmission in the ACC of MDD patients who completed suicide (Zhao et al., 2018). Interestingly, PACAP was previously found to increase GABA neurotransmission in the central amygdala (Varodayan et al., 2020). The ACC has been shown to be related to the emotional reaction to intense psychological pain rather than to the perception of pain itself (Price, 2000). Therefore, the hyperactivity of the ACC in which PACAP might take part may be related to psychological pain in suicide.

#### Relation between PACAP and CRH in the hypothalamus and PFC

In AD patients we observed a positive correlation between the Cornell depression score and PACAP-ir in the PVN, while in the same cohort we observed earlier a positive correlation between, the Cornell depression score and CRH in the PVN (Meynen et al., 2007). This suggests a stimulating effect of PACAP on the hypothalamic CRH neurons in AD depression. Moreover, PACAP-driven hypothalamic CRH may be involved in suicide risk (Berardelli et al., 2020). Some observations suggest that a similar relationship may exist between PACAP and CRH within the PFC. Earlier we found an increased expression of CRH-mRNA in the ACC of MDD patients following suicide (Zhao et al., 2015). Interestingly, these alterations correlated with increased expression levels of glutamate and GABA related genes in the ACC in suicidal MDD patients (Zhao et al., 2018). Now we have observed an increased PACAP-mRNA expression in the ACC of MDD patients following suicide. Also Pandey, Rizavi, Bhaumik, and Ren (2019) found that CRH levels were higher in depressed suicide victims, not only in the hypothalamic PVN, but also in the PFC in MDD, which was related to the severity of the symptoms. It should be noted, however, that in contrast with these observations some previous studies (López, Little, & Watson, 2000; Nemeroff, Owens, Bissette, Andorn, & Stanley, 1988) found downregulated CRH in the PFC of suicidal patients.

#### mRNA Expression alterations in PACAP and its receptor genes in BD and MDD in relation to psychotic features

Knock-out mice with disruption of the PACAP gene are vulnerable to ‘psychotic-like features’, such as exaggerated head-twitch responses and sensorimotor gating deficits (Hazama et al., 2014). We observed in the ACC and DLPFC, that VPAC1-mRNA and VPAC2-mRNA expression were significantly increased in BD psychotic patients compared to BD patients without psychosis. In addition, we observed increased PACAP-mRNA expression in the DLPFC in MDD patients with psychotic features compared to MDD patients without psychosis. However, Hashimoto et al. (2007) found seemingly decreased PACAP and increased PAC1-mRNA in the PFC of rodents with psychotic features. This makes clear that there is a need for validation of rodent models in relation to the human observations.

### Sex differences

We found more PACAP-ir in the PVN of female controls than in matched male controls. One may speculate that this sex difference may be related to the higher vulnerability of women to mood disorders, possibly by the stimulation of PACAP expression by oestrogens, as appeared from the analysis of single nucleotide polymorphisms spanning the PACAP and PAC1 genes (Ressler et al., 2011). The oestrogenic response element SNP (rs2267735), which is associated with post-traumatic stress disorder in women, is involved in PAC1 gene regulation (Ressler et al., 2011). Moreover, studies in rats have shown that oestrogens induced PAC1 expression (Ressler et al., 2011). Concerning our observation of a sex difference in controls, it should be noted, however, the great majority donors were in postmenopausal women. Therefore, the stimulation of PVN PACAP-ir may in that case come from neurosteroids produced locally in the brain.

No sex differences were reported in mRNA expression of PACAP and its receptors in the PFC, which is consistent with the absence of sex differences in VPAC1 or VPAC2 receptors in the rat PFC (Shneider, Shtrauss, Yadid, & Pinhasov, 2010).

### pH, Agonal state and endophenotype

The agonal process, which is the irreversible decomposition of vital constants up to the moment of death, can influence the amount and quality of mRNA. Lower pH levels were found throughout the brain in cases of death following a protracted illness such as respiratory distress, compared with sudden death cases. Brain pH does not differ significantly across the regions. Subjects who died after a long terminal illness have a lower pH in the brain, CSF and blood, while people who died suddenly by an accident generally have a pH around 7. The acidosis corresponds with the increased lactic acid concentrations. Agonal factors have an impact on RNA integrity (RIN) and gene expression profiles. pH>6.0, as was present in our samples, is a good criterion to ensure RNA quality. Moreover, we have carefully matched the samples for pH. Many studies have indicated that postmortem brain pH is a fair indication of mRNA preservation as indicated by the RIN value. There is no significant change in the pH within a postmortem delay of 24 h (for review see Swaab & Bao, 2021). Since the CSF pH was well matched in our hypothalamic samples between MDD (*p* = 0.66), BD (*p* = 0.79) and AD (*p* = 0.30) donors and their respective controls, and of the MDD cortex samples and their controls (*p* = 0.65), the agonal state of these patients has not influenced our data.

However, it should be noted that there are conditions, such as BD, where a little lower CSF pH is not considered a confounding factor, but rather an endophenotype of this disorder (Dogan, Yuksel, Du, Chouinard, & Öngür, 2018; Hagihara et al., 2018; Slabe et al., 2023). This phenomenon was also observed in the present study, and is due to increased lactate levels (Dogan et al., 2018; Hagihara et al., 2018). It and has also been found *in vivo* by magnetic resonance spectroscopy, especially in the cingulate cortex and frontal lobe of BD patients (Dogan et al., 2018; Hagihara et al., 2018). The RIN value did not show a significant difference between BD patients and controls (see online Supplementary Table S9b). The small difference in pH in BD samples has thus not affected our qPCR data.

Brain pH is also associated with changes in gene expression, and therefore presents a potential biomarker that should be examined further in relation to neuropsychiatric disorders (Dogan et al., 2018; Slabe et al., 2023). We observed a positive correlation between the mRNA expression between PACAP, VPAC1 and VPAC2 and CSF pH in the ACC of BD patients, and between PACAP-mRNA and pH in the ACC of BD-P patients. This means that the lower CSF pH in BD may counteract the effects of PACAP-related molecules. In relation to suicide, there were negative correlations found between CD38, PAC1 and pH in those who died of natural causes, indicating that the lower pH in BD may increase the sensitivity of the ACC to PACAP and thus to suicide.

### Limitations

We matched the mood disorder patients and controls for as many factors as possible. One of the unavoidable possible confounding factors in a post-mortem study is the medication used. The only information for this putative confounder that we have for the PFC study is fluphenazine. Previous *in vitro* research has found decreased PAC1-mRNA expression after incubation with the typical neuroleptic haloperidol, which was also used by some of our patients, while all the examined neuroleptics decrease VPAC2 mRNA expression (Jóźwiak-Bębenista & Kowalczyk, 2017). It has been proposed, therefore, that PACAP receptors may be involved in the mechanism of typical and atypical neuroleptic drugs (Jóźwiak-Bębenista & Kowalczyk, 2017). This effect may not explain why we detected increased VPAC2 and VPAC1 mRNA expression in patients with psychotic features. Reichenstein, Rehavi, and Pinhasov (2008) showed that selective serotonin reuptake inhibitors in cortical neuron-enriched cultures suppress PAC1-mRNA and VPAC2-mRNA expression and upregulate PACAP-mRNA expression, while tricyclic antidepressants (TCA) have an opposite effect. Furthermore, benzodiazepines, which are known to increase PACAP-mRNA in cortical areas (Tamaki et al., 2008), were administered to some of our subjects, and may have contributed to some of the increases we observed in PACAP.

For the hypothalamic studies we excluded patients who had received corticosteroid therapy during the three months prior to death because of its effect on the human hypothalamic stress systems (Erkut, Pool, & Swaab, 1998) and the relationship between PACAP and CRH (see above). Unfortunately, there is no information on the effect of other medicines the patients used on PACAP or its receptors in the human hypothalamus and PFC. It is, however, reassuring that research in animal stress models has shown that PACAP may play a role in the regulation of the HPA axis in response to psychogenic stress (Eiden, 2013; Lehmann et al., 2013) and in the regulation of the sympathetic nervous system (Hashimoto et al., 2011).

## Conclusions

Higher hypothalamic PACAP-ir was present in the PVN in control women compared to men, in BD men compared to both, BD women and to MDD men, and in AD patients with depression compared to AD patients without depression. The mRNA alterations in the PFC of PACAP and were differential depending on the type of mood disorder and brain area. Increased PACAP-mRNA expression was found in the ACC and DLPFC in BD and MDD patients with accomplished suicide, as compared to BD patients who died from natural causes. Increased expression of PACAP-related genes in relation to psychotic features was found in the DLPFC and ACC in both MDD and BD patients compared to mood disorder patients without psychotic features. These observations suggest a potential beneficial effect of PACAP antagonists. However, PACAP is widely distributed across the brain and changes in different directions in different brain areas in mood disorders. Since PACAP is a highly multifunctional peptide involved in many processes (Pinhasov et al., 2011; Zhang et al., 2021), the effects of administration to patients cannot be predicted. This problem is illustrated by the observations in a three-hit animal model for depression, where PACAP had even antidepressant effects (Farkas et al., 2017; Gaszner et al., 2022). Whether PACAP antagonists or agonists (Hashimoto et al., 2016; Pinhasov et al., 2011) may be potential novel therapeutics in mood disorders, especially in suicidal behaviour or psychotic features, needs further clinical studies.

## Supporting information

Slabe et al. supplementary material Slabe et al. supplementary material

## Supplementary material

The supplementary material for this article can be found at https://doi.org/10.1017/S0033291723001265.
