# Supplementary material for: Alterations in pituitary adenylate cyclase-activating polypeptide in major depressive disorder, bipolar disorder, and comorbid depression in Alzheimer's disease in the human hypothalamus and prefrontal cortex
Source: Psychol Med. 2023 May 25;53(16):7537–49. doi: 10.1017/S0033291723001265 (PMC10755247; doi:10.1017/S0033291723001265)
Supplement: Slabe et al. supplementary material [file S0033291723001265sup001.doc]

**Supplementary material**

**Supplementary (S) Tables**

**Table S1**: Clinico-pathological information of NBB donors for hypothalamus study mood disorders

| **NBB** | **Group** | **Sex** | **Age (y)** | **PMD (hr:min)** | **FT (d)** | **CTD** | **MOD** | **CSF pH** | **BW (g)** | **Braak stage** | **Medication in the past** | **Medication in the last 3 months** | **Suicide attempt** |
| --- | --- | --- | --- | --- | --- | --- | --- | --- | --- | --- | --- | --- | --- |
| 98-010 | BD | F | 75 | 4:00 | 38 | 20:45 | 1 | ND | 1123 | 1 | TeCA, TCA | BZD, TeCA, SSRI, Hal, Mo | No |
| 02-014 | BD | M | 68 | 12.00 | 30 | 0:00 | 2 | 6.64 | 1414 | 1 | Li, Hal, ZUC, MAOI | MAOI | No |
| 99-118 | BD | M | 68 | 5:55 | 33 | 23:15 | 10 | 6.82 | 1174 | 1 | Li, SSRI | Li | No |
| 00-111 | BD | M | 70 | 4:50 | 43 | 2:45 | 10 | 6.26 | 1442 | 1 | Li, ZUC, valproate, BZD,ECT | Li, ECT | No |
| 00-088 | BD | M | 73 | 5:15 | 36 | 9:30 | 7 | 6.38 | 1145 | 2 | Li, BZD, SSRI, Hal, ECT, MAOI, Methylphenidate | Li, SSRI, Hal, Methylphenidate | No |
| 12-048 | BD | M | 81 | 6:40 | 60 | 20:00 | 5 | 6.7 | 1283 | 2 | Li, prednisolone | Li, BZD, amitriptyline, valproate | No |
| 12-110 | BD | M | 87 | 3:15 | 53 | 23:00 | 10 | 6.39 | 1285 | 3 | BZD, valproate | BZD, Mo, valproate | No |
| 92-003 | MDD | F | 55 | 6:45 | 52 | 7:45 | 11 | 6.4 | 1320 | ND | BZD, SSRI, TeCA | SSRI, BZD, | Yes |
| 94-017 | MDD | F | 72 | 22:00 | 39 | 19:00 | 1 | ND | 1287 | ND | TeCA, BZD, prednisone | TeCA, BZD, prednisone | No |
| 12-097 | MDD | F | 73 | 5:45 | 61 | 15:30 | 9 | 6.7 | 1205 | 3 | TeCA | TCA,TeCA, BZD, pancuronium, Ba | No |
| 08-076 | MDD | F | 91 | 5:20 | 36 | 9:05 | 8 | 6.53 | 1163 | 3 | Hal, SSRI, BZD, levothyroxine | SSRI, BZD, levothyroxine | No |
| 08-031 | MDD | F | 93 | 4:20 | 51 | 4:55 | 3 | 6.8 | 1023 | 2 | SSRI, BZD | Mo, BZD, SSRI | Yes |
| 94-032 | MDD | M | 71 | 16:15 | 38 | 16:15 | 2 | ND | 975 | ND | ZUC, BZD, MAOI, clomipramine | None | Yes |
| 95-036 | MDD | M | 74 | 62:55:00 | 35 | 17:05 | 3 | ND | 1444 | ND | SSRI, BZD, cisordinol | SSRI, BZD, cisordinol | Yes |
| 02-051 | MDD | M | 81 | 6:00 | 34 | 15:30 | 6 | 6.5 | 1280 | 3 | None | Hal | No |
| 11-058 | MDD | M | 83 | 10:40 | 57 | 5:00 | 7 | 6.5 | 1200 | 2 | TCA, TeCA, Hal, Pipamperon, SSRI | BZD, gabapentin, Mo | No |
| 07-060 | MDD | M | 93 | 6:00 | 49 | 21:10 | 9 | 6.37 | 1369 | 1 | Paroxetine, BZD, TCA, fluoxetine, paroxetine | BZD |  |
| Median |  |  | 74 |  | 39 |  | 7 | 6.5 | 1280 | 2 |  |  |  |

Note: BD: bipolar disorder; Braak stage: progression of pathological changes for Alzheimer’s disease according to Braak & Braak (1991); BW: brain weight; BZD: benzodiazepine; CSF pH: cerebrospinal fluid pH; CTD: clock time at death; CTR: control; F: female; FT(d): fixation time in days; Hal: haloperidol; Li: lithium; M: male; MDD: major depressive disorder; MOD: month of death; NBB: Netherlands Brain Bank; ND: no data; PMD: post-mortem delay; SSRI: selective serotonin reuptake inhibitors; TCA: tricyclic antidepressants; TeCA: tetracyclic antidepressants.

**Table S2a**: Clinico-pathological information of NBB donors for hypothalamus study controls

| **NBB** | **Group** | **Sex** | **Age (y)** | **PMD (hr:min)** | **FT (d)** | **MOD** | **CSF ph** | **BW (g)** | **Braak stage** | **Medication in the past** | **Medication in the last 3 months** |
| --- | --- | --- | --- | --- | --- | --- | --- | --- | --- | --- | --- |
| 99-044 | CTR | F | 88 | 05:55 | 34 | 4 | 6.05 | 1115 | 1 | BZD | BZD |
| 97-042 | CTR | F | 65 | 12:50 | 28 | 4 | 6.94 | 910 | 1 | None | Adrenalin, dopamine |
| 01-069 | CTR | F | 68 | 05:45 | 32 | 5 | 6.97 | 1135 | 1 | None | None |
| 14-020 | CTR | F | 92 | 06:35 | 38 | 4 | 6.12 | 1305 | 3 | None | Adrenalin, dopamin |
| 12-094 | CTR | F | 82 | 05:15 | 47 | 8 | 6.34 | 1221 | 2 | BZD | None |
| 08-105 | CTR | F | 89 | 03:52 | 58 | 12 | 7.3 | 1258 | 3 | BZD, prednisolone, βB | Hal, morphine |
| 14-051 | CTR | M | 92 | 07:45 | 47 | 9 | 6.55 | 1210 | 3 | BZD, βB | BZD |
| 90-080 | CTR | M | 85 | 04:55 | 28 | 11 | 6.34 | 1035 | 3 | None | None |
| 15-033 | CTR | M | 93 | 07:40 | 65 | 4 | 6.2 | 1155 | 0 | None | BZD, morphine |
| 92-049 | CTR | M | 71 | 05:40 | 32 | 4 | 7.4 | 1250 | ND | None | None |
| 00-072 | CTR | M | 78 | 18:00 | 45 | 6 | 5.84 | 1172 | 1 | Isomeride, Hal, clonazepam | Hal, diazepam, morphine |
| 18-021 | CTR | M | 92 | 08:50 | 62 | 2 | 6.89 | 1245 | 3 | None | Morphine |
| 06-028 | CTR | M | 76 | 19:35 | 27 | 4 | 6.5 | 1494 | 3 | Homeopathy | None |
| 09-075 | CTR | M | 88 | 07:00 | 44 | 10 | 6.76 | 1230 | 3 | βB | βB |
| 09-001 | CTR | M | 88 | 04:43 | 51 | 1 | 6.17 | 1418 | 2 | prednisolone | Hal, digoxin, Mo |
| 98-055 | CTR | M | 85 | 21:20 | 31 | 4 | ND | 1290 | ND | None | None |
| 09-003 | CTR | M | 62 | 07:20 | 47 | 1 | 6.36 | 1520 | 1 | None | None |
| Median |  |  | 85 | 7:00 | 44 | 4 | 6.43 | 1230 | 2 |  |  |

Note: βB: beta blocker; BD: bipolar disorder; Braak stage: progression of pathological changes for Alzheimer’s disease according to Braak & Braak (1991); BW: brain weight; BZD: benzodiazepine; CSF pH: cerebrospinal fluid pH; CTD: clock time at death; CTR: control; F: female; FT(d): fixation time in days; Hal: Haloperidol; M: male; MOD: month of death; NBB: Netherlands Brain Bank; ND: no data.

**Table S2b. p-values for matching for confounding factors for mood disorders study**

| **Confounding factor** | **C M vs BD M** | **MDD M vs C M** | **C M vs C F** | **MDD+BD vs C** |
| --- | --- | --- | --- | --- |
| Age | 0.07 | 0.56 | 0.79 | 0.18 |
| PMD | 0.12 | 0.57 | 0.18 | 0.57 |
| Ft | 0.94 | 0.89 | 0.71 | 0.47 |
| CTD | 0.51 | 0.19 | 0.87 | 0.12 |
| MOD | 0.19 | 0.84 | 0.43 | 0.66 |
| pH | 0.64 | 0.94 | 0.81 | 0.64 |
| BW | 0.88 | >0.9999 | 0.26 | 0.81 |
| Braak | 0.56 | 0.90 | 0.58 | 0.73 |
|  | **BD vs C** | **MDD vs C P value** | **MDD M vs MDD F** | **MDD+BD F vs C F** |
| Age | 0.07 | 0.61 | 0.75 | 0.94 |
| PMD | 0.11 | 0.68 | 0.21 | 0.97 |
| Ft | 0.96 | 0.31 | 0.31 | 0.20 |
| CTD | 0.20 | 0.23 | 0.33 | 0.31 |
| MOD | 0.54 | 0.89 | 0.69 | 0.61 |
| pH | 0.79 | 0.66 | 0.20 | >0.9999 |
| BW | 0.71 | 0.98 | 0.69 | 0.82 |
| Braak | 0.32 | 0.60 | 0.70 | 0.75 |
|  | **MDD F vs C F** | **MDD vs BD P value** | **MDD M vs BD M** | **MDD+BD M vs C M** |
| Age | >0.9999 | 0.30 | 0.19 | 0.13 |
| PMD | 0.83 | 0.08 | 0.07 | 0.51 |
| Ft | 0.18 | 0.52 | 0.93 | 0.99 |
| CTD | 0.54 | 0.52 | >0.9999 | 0.22 |
| MOD | 0.97 | 0.76 | 0.31 | 0.34 |
| pH | >0.9999 | 0.86 | 0.67 | 0.7 |
| BW | 0.66 | 0.89 | 0.93 | 0.95 |
| Braak | 0.27 | 0.19 | 0.71 | 0.60 |

Note: BD: bipolar disorder; Braak: progression of pathological changes for Alzheimer’s disease according to Braak & Braak (1991); BW: brain weight; pH: pH of cerebrospinal fluid; C: controls; CTD: clock time at death; F: females; Ft(d): fixation time in days; M: males; MDD: major depressive disorder; MOD: month of death; PMD: post-mortem delay.

**Table S3a**: Clinico-pathological information of NBB Alzheimer donors with or without Depression

| **NBB** | **Braak** | **CS** | **SEX** | **AGE** | **PMD** | **Ft (d)** | **Depression status** |
| --- | --- | --- | --- | --- | --- | --- | --- |
| 97-087 | 5 | 2 | F | 78 | 3.17 | 33 | AD, No current Depression |
| 93-030 | 5 | 2 | F | 87 | 3 | 32 | AD, No current Depression |
| 99-083 | 5 | 4 | F | 90 | 3.5 | 29 | AD, No current Depression |
| 93-040 | 4 | 4 | M | 83 | 3.25 | 26 | AD, No current Depression |
| 97-112 | 5 | 5 | F | 85 | 4.58 | 32 | AD, No current Depression |
| 98-142 | 6 | 5 | F | 78 | 5 | 39 | AD, No current Depression |
| 92-091 | 4 | 5 | F | 94 | 3.83 | 32 | AD, No current Depression |
| 94-071 | 5 | 5 | F | 96 | 5.75 | 35 | AD, No current Depression |
| 94-029 | 4 | 8 | F | 83 | 4.92 | 36 | AD, No current Depression |
| 93-087 | 5 | 8 | M | 81 | 4.17 | 137 | AD, No current Depression |
| 95-089 | 4 | 9 | F | 89 | 4.58 | 30 | AD, No, current Depression |
| 92-099 | 6 | 9 | F | 66 | 5.5 | 44 | AD, No current Depression |
| 96-068 | 6 | 9 | F | 82 | 3.17 | 28 | AD, No current Depression |
| 94-012 | 6 | 10 | M | 64 | 3.67 | 30 | AD, No current Depression |
| 94-110 | 6 | 11 | F | 82 | 5 | 11 | AD, Depression |
| 95-038 | 6 | 12 | F | 85 | 1.58 | 32 | AD, Depression |
| 94-101 | 6 | 14 | F | 87 | 5 | 33 | AD, Depression |
| 96-029 | 5 | 14 | F | 78 | 4.5 | 34 | AD, Depression |
| 93-113 | 4 | 17 | M | 91 | 6 | 31 | AD, Depression |
| 93-008 | 5 | 18 | F | 69 | 4 | 33 | AD, Depression |
| 93-050 | 6 | 18 | F | 80 | 2.75 | 122 | AD, Depression |
| 93-026 | 6 | 25 | M | 76 | 4.75 | 32 | AD, Depression |
| 92-083 | 4 | 26 | M | 82 | 4.25 | 32 | AD, Depression |
| 93-060 | Could not be scored | | M | 79 | 3 | 53 | Control |
| 95-016 | 2 |  | F | 86 | 13.5 | 30 | Control |
| 96-078 | 2 |  | F | 87 | 8 | 31 | Control |
| 97-008 | 1 |  | F | 88 | 3.75 | 30 | Control |
| 97-156 | 1 |  | F | 77 | 2.67 | 47 | Control |
| 99-116 | 0 |  | M | 78 | 4.33 | 43 | Control |
| 12-094 | 2 |  | F | 82 | 5.15 | 47 | Control |
| 90-080 | 3 |  | M | 85 | 4.92 | 28 | Control |
| 90-001 | 2 |  | M | 88 | 4.72 | 51 | Control |
| Median |  |  |  | 82.5 | 4.42 | 32 |  |

Note: Braak stage: progression of pathological changes for Alzheimer’s disease according to Braak & Braak (1991); AD, Alzheimer’s disease; BW: brain weight; CTD: clock time at death; CS: Cornell score (Alexopoulos, Abrams, Young, & Shamoian, 1988); CTR: control; F: female; Ft(d): fixation time in days; M: male; MOD: month of death; NBB: Netherlands Brain Bank; PMD: post-mortem delay. For clinical details see (Hoogendijk et al., 1999; Meynen, Unmehopa, Hofman, Swaab, & Hoogendijk, 2007).

**Table S3b**. p-values for matching for confounding factors for study on Alzheimer’s disease with or without depression

| **Confounding factors** | **CTR vs AD+ADDe** | **CTR vs ADDe** | **CTR vs AD** | **AD vs ADDe** |
| --- | --- | --- | --- | --- |
| Age | 0.75 | 0.43 | 0.95 | 0.5 |
| PMD | 0.48 | 0.66 | 0.47 | 0.68 |
| FT(d) | 0.42 | 0.76 | 0.34 | 0.99 |
| pH | 0.30 | 0.43 | 0.33 | 0.64 |
| Braak | 2,30E-05 | 0.0005 | 0.0001 | 0.67 |

Note: AD: Alzheimer patients, no depression; ADDe: Alzheimer’s patients with depression; BS: Braak stage- progression of pathological changes for Alzheimer’s disease according to Braak & Braak (1991); Ft(d): fixation time in days; MOD: month of death; NBB: Netherlands Brain Bank; No = no depression; PMD: post-mortem delay.

**Table S4a:** Clinico-pathological information and matching SMRI Depression Collection

| **DC** | **MDD** | **Ctr** | **p-value** |
| --- | --- | --- | --- |
| Age (year)^1^ | 42 (24-63) | 49 (24-63) | 0.233 |
| Gender (F/M) | 11/13 | 4/8 | 0.473^2^ |
| PMD (hour)^1^ | 25.5 (13-65) | 28 (9-40) | 0.534 |
| Brain pH^1^ | 6.63 (6.3-6.9) | 6.60 (6.31-6.91) | 0.649 |
| Brain weight (g)^1^ | 1460 (1170-1780) | 1465 (1200-1595) | 0.876 |
| Hemisphere | 15L/9R | 6L/6R | 0.473^2^ |
| Age of onset (year)^1^ | 30 (13-59) |  |  |
| Duration of illness (year)^1^ | 10.50 (0.1-31) |  |  |
| Suicide | 17 |  |  |
| Psychotic features | 12 |  |  |

Note: Ctr: control; DC: depression collection; F: women; L: left; M: men; MDD: major depressive disorder; L: left; PMD: post-mortem delay; SMRI: The Stanley Medical Research Institute; R: right.

^1^ Data showed with median range

^2^ Chi-square test

**Table S4b**. p-values for matching of confounding factors of SMRI donors of Depression collection for prefrontal cortex study

| **Confounding factors** | **Ctr-F vs Ctr-M** | **MDD-F vs MDD-M** | **MDD-NP-F vs MDD-NP-M** | **MDD-P-F vs MDD-P-M** | **MDD-S-F vs MDD-P-M** | **MDD-NS-F vs MDD-NS-M** |
| --- | --- | --- | --- | --- | --- | --- |
| Age | 0.48 | 0.97 | 0.56 | 0.78 | 0.79 | >0.9999 |
| PMD | 0.48 | 0.47 | 0.26 | 0.79 | 0.35 | >0.9999 |
| PH | 0.65 | 0.52 | 0.46 | 0.51 | 0.69 | 0.2 |
| BW | **0.03** | **0.002** | **0.048** | **0.03** | **0.03** | 0.1 |
| Hemipshere | 0.22 | 0.07 | 0.20 | 0.22 | **0.04** | >0.9999 |
|  | **Ctr vs MDD-P** | **Ctr vs MDD-NP** | **MDD-P vs MDD-NP** | **Ctr vs MDD-S** | **Ctr vs MDD-NS** | **MDD-S vs MDD-NS** |
| Age | 0.30 | 0.32 | 0.72 | 0.12 | 0.84 | 0.25 |
| PMD | 0.09 | 0.54 | **0.02** | 0.61 | 0.80 | 0.96 |
| PH | 0.77 | 0.27 | 0.10 | 0.40 | 0.98 | 0.57 |
| BW | 0.79 | >0.9999 | 0.80 | 0.77 | 0.87 | 0.86 |
| Hemipshere | 0.81 | 0.81 | 0.67 | 0.81 | 0.85 | 0.74 |
| Gender | 0.41 | 0.67 | 0.68 | 0.67 | 0.49 | 0.71 |

Note: BW: brain weight; pH: cerebrospinal fluid pH; Ctr: controls; M: males; MDD; Major depressive disorder; NP: without psychotic features; NS; patients that did not died of suicide; P: patients with psychotic features; PMD: post-mortem delay S; patients who completed suicide; SMRI: The Stanley Medical Research Institute.

**Table S5a**: Clinico-pathological information and matching SMRI Array collection

| **AC** | **BD** | **Ctr** | **p-value** |
| --- | --- | --- | --- |
| Age (year)^1^ | 44 (19-64) | 45 (31-60) | 0.66 |
| Gender (M/F) | 15/15 | 25/9 | 0.058^2^ |
| PMD (hour)^1^ | 28.5 (12-84) | 30 (9-58) | 0.17 |
| Brain pH^1^ | 6.50 (5.92-6.97) | 6.69 (6.00-7.03) | 0.027 |
| Brain weight (g)^1^ | 1420 (1170-1670) | 1412.5 (1120-1900) | 0.49 |
| Hemisphere | 14L/17R | 16L/18R | 0.44^2^ |
| Age of onset (year)^1^ | 22.5 (14-48) |  |  |
| Duration of illness (year)^1^ | 18 (2-45) |  |  |
| Suicide | 13 |  |  |
| Psychotic features | 16 |  |  |

Note: AC: Array collection; Ctr: control; F: women; L: left; M: men; BD: bipolar disorder; L: left; PMD: post-mortem delay; SMRI: The Stanley Medical Research Institute; R: right.

^1^ Data showed with median range

^2^ Chi square test

**Table 5b**. p-values for matching of confounding factors of SMRI ARRAY collection for mood disorder prefrontal cortex study

| **Confounding factor** | **C-F vs C-M** | **BD-F vs BD-M** | **BD-NP-F vs BD-NP-M** | **BD-P-F vs BD-P-M** | **BD-S-F vs BD-S-M** | **BD-NS-F vs BD-NS-M** |
| --- | --- | --- | --- | --- | --- | --- |
| Age | 0.06 | 0.62 | 0.32 | 0.78 | 0.51 | 0.83 |
| PMD | 0.32 | 0.35 | 0.88 | **0.02** | 0.91 | 0.16 |
| PH | 0.09 | 0.85 | 0.53 | 0.39 | 0.51 | 0.56 |
| BW | **<0.0001** | **0.0002** | **0.03** | **0.01** | **0.01** | **0.002** |
| Hemipshere | 0.10 | 0.07 | 0.41 | 0.38 | 0.85 | **0.0071** |
|  | **C vs BD-S** | **C vs BD-NS** | **BD-S vs BD-NS** | **C vs BD-P** | **C vs BD-NP** | **BD-P vs BD-NP** |
| Age | 0.78 | 0.68 | 0.86 | 0.95 | 0.50 | 0.76 |
| PMD | 0.1 | 0.37 | 0.45 | 0.13 | 0.27 | 0.95 |
| PH | 0.2 | **0.028** | 0.53 | **0.005** | 0.61 | 0.09 |
| BW | 0.18 | **0.03** | **0.006** | 0.45 | 0.97 | 0.69 |
| Hemipshere | 0.17 | >0.9999 | 0.22 | 0.83 | 0.24 | 0.35 |
| Gender | 0.20 | 0.06 | 0.71 | **0.04** | 0.33 | 0.45 |

Note: BD: bipolar disorder; BW: brain weight; pH: cerebrospinal fluid pH; C: controls; F: females; FT(d): fixation time in days; M: males; NP: without psychotic features; NS; patients that did not died of suicide; P: patients with psychotic features; PMD: post-mortem delay S; patients who completed suicide.

**Table S6**: Clinico-pathological information control patients of SMRI Array collection

| **Group** | **Age** | **Sex** | **Cause of death** | **PMI** | **BrainPH** | **Brain**  **Weight** | **RIN** | |
| --- | --- | --- | --- | --- | --- | --- | --- | --- |
|  |  |  |  |  |  |  | **DLPFC** | **ACC** |
| CTR1 | 44 | 2 | CARDIAC | 28 | 6.59 | 1330 | 8.3 | 7.5 |
| CTR2 | 49 | 1 | CARDIAC | 46 | 6.5 | 1605 | 8.3 | 7.5 |
| CTR3 | 53 | 1 | CARDIAC | 9 | 6.4 | 1500 | 9.1 | 8 |
| CTR4 | 37 | 1 | CARDIAC | 13 | 6.5 | 1600 | 8.3 | 7.8 |
| CTR5 | 51 | 1 | CARDIAC | 31 | 6.7 | 1400 | 7.3 | 7.6 |
| CTR6 | 53 | 1 | CARDIAC | 28 | 6 | 1340 | 8.4 | 5.2 |
| CTR7 | 38 | 2 | CARDIAC | 33 | 6 | 1120 | 9.7 | 7.3 |
| CTR8 | 38 | 2 | CARDIAC | 28 | 6.7 | 1350 | 8.8 | 7.6 |
| CTR9 | 60 | 1 | CARDIAC | 47 | 6.8 | 1460 | 8.4 | 6.9 |
| CTR10 | 35 | 1 | MYOCARDITIS | 52 | 6.7 | 1700 | 8.7 | 8 |
| CTR11 | 34 | 1 | CARDIAC | 22 | 6.48 | 1480 | 8.2 | 7.7 |
| CTR12 | 47 | 1 | CARDIAC | 21 | 6.81 | 1550 | 8.7 | 7.5 |
| CTR13 | 45 | 1 | CARDIAC | 29 | 6.94 | 1405 | 8.5 | 8.6 |
| CTR14 | 34 | 2 | CARDIAC | 24 | 6.87 | 1255 | 6.6 | 8.8 |
| CTR15 | 42 | 1 | CARDIAC | 37 | 6.91 | 1340 | 7.8 | 8.4 |
| CTR16 | 44 | 2 | CARDIAC | 10 | 6.2 | 1305 | 8.4 | 5.6 |
| CTR17 | 45 | 1 | CARDIAC | 18 | 6.81 | 1585 | 9.5 | 8.8 |
| CTR18 | 49 | 1 | CARDIAC | 23 | 6.93 | 1390 | 8.3 | 8.7 |
| CTR19 | 32 | 1 | CARDIAC | 24 | 7.03 | 1415 | 8.3 | 7.5 |
| CTR20 | 55 | 1 | CARDIAC | 31 | 6.7 | 1515 | 7.9 | 7.5 |
| CTR21 | 49 | 2 | CARDIAC | 45 | 6.72 | 1435 | 8.3 | 8.8 |
| CTR22 | 33 | 2 | ASTHMA | 29 | 6.52 | 1360 | 7 | 6.5 |
| CTR23 | 48 | 1 | CARDIAC | 31 | 6.86 | 1580 | 8 | 7.5 |
| CTR24 | 50 | 1 | CARDIAC | 49 | 6.75 | 1645 | 7.5 | 8 |
| CTR25 | 32 | 1 | CARDIAC | 13 | 6.57 | 1410 | 8.3 | 8.9 |
| CTR26 | 47 | 1 | CARDIAC | 11 | 6.6 | 1495 | 8.6 | 9 |
| CTR27 | 46 | 1 | CARDIAC | 31 | 6.67 | 1360 | 8.7 | 9.1 |
| CTR28 | 40 | 1 | CARDIAC | 38 | 6.67 | 1498 | 8.7 | 8.1 |
| CTR29 | 51 | 1 | CARDIAC | 22 | 6.71 | 1900 | 8.4 | 7.8 |
| CTR30 | 31 | 1 | PULM EMBOL | 11 | 6.13 | 1335 | 8.6 | 5.2 |
| CTR31 | 48 | 1 | CARDIAC | 24 | 6.91 | 1321 | 8.4 | 6.4 |
| CTR32 | 39 | 2 | CARDIAC | 58 | 6.46 | 1260 | 8 | 5.8 |
| CTR33 | 47 | 1 | CARDIAC | 36 | 6.57 | 1535 | 7.3 | 6.8 |
| CTR34 | 41 | 2 | CARDIAC | 50 | 6.17 | 1290 | 6.6 | 6.4 |
| Median | 45 |  |  | 28.5 | 6.685 | 1412.5 | 8.3 | 7.6 |

Note: ACC: anterior cingulate cortex; CARDIAC: cardiac arrest; CTR: control; DLPFC: dorsolateral prefrontal cortex; PMI: post-mortem interval; PULM EMBOL: pulmonary embolism; RIN: RNA integrity number 1: male, 2: female.

**Table S7**: Clinico-pathological information Bipolar patients of SMRI Array collection

| **Group** | **DSM_IV** | **Age** | **Sex** | **Suicide Status** | **Cause of death** | **PMI** | **Brain PH** | **Brain Weight** | **Psychotic Feature** | **Lifetime Antipsychotics** | **RIN** | |
| --- | --- | --- | --- | --- | --- | --- | --- | --- | --- | --- | --- | --- |
|  |  |  |  |  |  |  |  |  |  |  | **DLPFC** | **ACC** |
| BD1 | 296.54 | 29 | 1 | 1 | SUIC:JUMPED | 48 | 6.39 | 1570 | 1 | 9000 | 9.1 | 8.3 |
| BD2 | 296.89 | 29 | 1 | 1 | SUIC:CO | 60 | 6.7 | 1430 | 0 | 0 | 7.8 | 7.6 |
| BD3 | 296.44 | 45 | 1 | 0 | CARDIAC | 28 | 6.35 | 1480 | 1 | 10000 | 8.3 | 9 |
| BD4 | 296.53 | 41 | 1 | 1 | SUIC:OD | 70 | 6.71 | 1625 | 0 | 0 | 8.4 | 9.1 |
| BD5 | 296.44 | 29 | 2 | 0 | OD | 62 | 6.74 | 1330 | 1 | 0 | 8.6 | 8.1 |
| BD6 | 296.53 | 44 | 1 | 1 | SUIC:HANGING | 19 | 6.74 | 1660 | 0 | 0 | 8 | 7.5 |
| BD7 | 296.44 | 49 | 2 | 1 | SUIC:MVA | 19 | 5.87 | 1380 | 1 | 4000 | 7.8 | 7.9 |
| BD8 | 296.7 | 48 | 2 | 0 | CARDIAC | 18 | 6.5 | 1205 | 0 | 0 | 7.3 | 8.4 |
| BD9 | 296.8 | 42 | 1 | 0 | DROWNING | 32 | 6.65 | 1470 | 0 | 0 | 9.5 | 8.3 |
| BD10 | 296.54 | 35 | 1 | 0 | CARDIAC | 35 | 6.3 | 1490 | 1 | 30000 | 8.9 | 8.6 |
| BD11 | 296.5 | 59 | 2 | 1 | SUIC:OD | 53 | 6.2 | 1410 | 0 | 0 | 7.3 | 6.2 |
| BD12 | 296.89 | 54 | 1 | 1 | SUIC:OD | 44 | 6.5 | 1510 | 0 | 0 | 8.2 | 7.8 |
| BD13 | 296.54 | 35 | 2 | 1 | SUIC:CO | 17 | 6.1 | 1250 | 1 | 3000 | 8.5 | 8.4 |
| BD14 | 296.64 | 42 | 2 | 0 | OD | 49 | 6.65 | 1335 | 1 | 15000 | 8.5 | 8.2 |
| BD15 | 296.54 | 58 | 2 | 1 | SUIC:GSW | 35 | 6.5 | 1440 | 1 | 12000 | 8.7 | 6.2 |
| BD16 | 295.7 | 64 | 1 | 0 | PNEUMONIA | 16 | 6.1 | 1340 | 1 | 130000 | 6.3 | 5.5 |
| BD17 | 296.53 | 59 | 1 | 0 | SLEEP APNEA | 84 | 6.65 | 1300 | 0 | 500 | 7.9 | 5.5 |
| BD18 | 296.44 | 51 | 1 | 0 | CARDIAC | 23 | 6.67 | 1590 | 1 | 1200 | 8 | 8.4 |
| BD19 | 296.89 | 63 | 2 | 0 | CARDIAC | 32 | 6.97 | 1290 | 0 | 0 | 8.5 | 8.7 |
| BD20 | 296.74 | 44 | 2 | 0 | MYOCARDITIS | 37 | 6.37 | 1200 | 1 | 30000 | 7.5 | 8.5 |
| BD21 | 296.89 | 56 | 2 | 0 | DROWNING | 26 | 6.58 | 1170 | 0 | 25000 | 9.3 | 7.9 |
| BD22 | 296.64 | 43 | 2 | 1 | SUIC:OD | 39 | 6.74 | 1505 | 1 | 4500 | 6.2 |  |
| BD23 | 296.74 | 35 | 1 | 0 | DROWNING | 22 | 6.58 | 1390 | 1 | 2000 | 8.5 | 7.4 |
| BD24 | 296.54 | 50 | 2 | 1 | SUIC:OD | 62 | 6.51 | 1400 | 1 | 15000 | 9.2 | 8.3 |
| BD25 | 296.54 | 49 | 2 | 0 | OD | 38 | 6.39 | 1190 | 1 | 0 | 7.9 | 7.2 |
| BD26 | 296.53 | 33 | 2 | 1 | SUIC:HANGING | 24 | 6.51 | 1450 | 0 | 3000 | 9.5 | 7.1 |
| BD27 | 296.72 | 41 | 2 | 0 | CARDIAC | 28 | 6.44 | 1360 | 0 | 3000 | 9.3 | 6.8 |
| BD28 | 296.8 | 43 | 2 | 0 | OD | 57 | 5.92 | 1340 | 1 | 10000 | 8.3 | 8.9 |
| BD29 | 296.54 | 56 | 1 | 1 | SUIC:OD | 23 | 6.07 | 1670 | 1 | 10000 | 8.6 | 6.9 |
| BD30 | 296.53 | 48 | 1 | 1 | SUIC:HANGING | 23 | 6.9 | 1466 | 0 | 0 | 8 | 7.6 |
| BD31 | 296.73 | 19 | 1 | 0 | OD | 12 | 5.97 | 1484 | 0 | 2000 | 8.4 | 8.6 |
| Median |  | 44 |  |  |  | 34 | 6.5 | 1420 |  |  | 8.4 | 8.1 |

Note: ACC: anterior cingulate cortex; BD: bipolar patient; CARDIAC: cardiac arrest; CO: carbon dioxide; DLPFC: dorsolateral prefrontal cortex; GSW: gunshot wound; MVA: motor vehicle accident death; OD: overdose; PMI: postmortem interval; Psychotic feature 0: without psychotic features; Psychotic feature 1: with psychotic features; RIN: RNA integrity number; Sex: 1 female, 2, male; SUIC: suicide.

**Table S8**: Clinico-pathological information SMRI Depression collection: DLPFC area

| **Group** | **Age** | **Sex** | **Psychotic** | **Suicide** | **PMI** | **PH** | **BW** | **RIN-DLPFC** |
| --- | --- | --- | --- | --- | --- | --- | --- | --- |
| CTR 1 | 34 | M | NO | NO | 9 | 6.56 | 1535 | 8.4 |
| CTR 2 | 50 | M | NO | NO | 11 | 6.5 | 1530 | 8.6 |
| CTR 3 | 24 | M | NO | NO | 17 | 6.6 | 1595 | 8 |
| CTR 4 | 50 | F | NO | NO | 35 | 6.31 | 1520 | 6.3 |
| CTR 5 | 39 | F | NO | NO | 24 | 6.88 | 1200 | 7.3 |
| CTR 6 | 56 | F | NO | NO | 29 | 6.78 | 1278 | 7.2 |
| CTR 7 | 48 | M | NO | NO | 12 | 6.51 | 1410 | 8.2 |
| CTR 8 | 44 | M | NO | NO | 27 | 6.82 | 1410 | 7.4 |
| CTR 9 | 35 | M | NO | NO | 31 | 6.59 | 1520 | 6.6 |
| CTR 10 | 63 | M | NO | NO | 40 | 6.91 | 1410 | 7.4 |
| CTR 11 | 56 | F | NO | NO | 31 | 6.66 | 1400 | 7.2 |
| CTR 12 | 63 | M | NO | NO | 37 | 6.5 | 1530 | 7.7 |
| MDD 1 | 53 | M | NO | NO | 21 | 6.64 | 1520 | 8.6 |
| MDD 2 | 56 | F | NO | NO | 15 | 6.59 | 1370 | 8.2 |
| MDD 3 | 44 | M | NO | NO | 24 | 6.52 | 1550 | 7.3 |
| MDD 4 | 45 | F | NO | NO | 29 | 6.9 | 1350 | 8.9 |
| MDD 5 | 36 | F | YES | NO | 32 | 6.74 | 1270 | 8.8 |
| MDD 6 | 40 | M | YES | NO | 52 | 6.48 | 1590 | 7.4 |
| MDD 7 | 45 | M | NO | YES | 29 | 6.75 | 1514 | 6.9 |
| MDD 8 | 24 | M | NO | YES | 21 | 6.61 | 1737 | 8.9 |
| MDD 9 | 45 | F | NO | YES | 13 | 6.58 | 1170 | 8.1 |
| MDD 10 | 33 | M | NO | YES | 25 | 6.86 | 1640 | 7.8 |
| MDD 11 | 56 | M | NO | YES | 38 | 6.59 | 1365 | 7.3 |
| MDD 12 | 32 | F | NO | YES | 19 | 6.8 | 1470 | 7.5 |
| MDD 13 | 34 | M | NO | YES | 24 | 6.79 | 1425 | 5.9 |
| MDD 14 | 47 | F | NO | YES | 25 | 6.88 | 1495 | 6.1 |
| MDD 15 | 28 | M | YES | YES | 26 | 6.7 | 1780 | 8 |
| MDD 16 | 32 | F | YES | YES | 19 | 6.7 | 1280 | 7.9 |
| MDD 17 | 48 | F | YES | YES | 24 | 6.36 | 1330 | 8 |
| MDD 18 | 63 | M | YES | YES | 31 | 6.6 | 1540 | 7.6 |
| MDD 19 | 40 | F | YES | YES | 49 | 6.72 | 1450 | 8.2 |
| MDD 20 | 35 | M | YES | YES | 19 | 6.6 | 1335 | 8 |
| MDD 21 | 28 | F | YES | YES | 40 | 6.68 | 1430 | 7.9 |
| MDD 22 | 35 | M | YES | YES | 36 | 6.6 | 1710 | 7.5 |
| MDD 23 | 62 | M | YES | YES | 65 | 6.57 | 1490 | 7.6 |
| MDD 24 | 51 | F | YES |  | 36 | 6.3 | 1440 | 7.1 |
| Median | 44.5 |  |  |  | 26.5 | 6.6 | 1460 | 7.65 |

Note: BW: brain weight; CTR: control; DLPFC: dorsolateral prefrontal cortex; F: female; M: male; MDD: major depressive disorder; PMI: post-mortem interval; RIN: RNA integrity number.

**Table S9a:** Clinico-pathological information SMRI Depression collection: ACC area

| **Group** | **Age** | **Sex** | **Suicide** | **Psychotic** | **PMI** | **PH** | **BW** | **Lifetime antipsychotics** | **RIN-ACC** |
| --- | --- | --- | --- | --- | --- | --- | --- | --- | --- |
| CTR 1 | 48 | M | NO | NO | 12 | 6.51 | 1410 | 0 | 8 |
| CTR 2 | 50 | F | NO | NO | 35 | 6.31 | 1520 | 0 | 6.3 |
| CTR 3 | 50 | M | NO | NO | 11 | 6.5 | 1530 | 0 | 7.7 |
| CTR 4 | 63 | M | NO | NO | 37 | 6.5 | 1530 | 0 | 7.9 |
| CTR 5 | 24 | M | NO | NO | 17 | 6.6 | 1595 | 0 | 8.3 |
| CTR 6 | 44 | M | NO | NO | 27 | 6.82 | 1410 | 0 | 7.6 |
| CTR 7 | 35 | M | NO | NO | 31 | 6.59 | 1520 | 0 | 7.9 |
| CTR 8 | 63 | M | NO | NO | 40 | 6.91 | 1410 | 0 | 7.8 |
| CTR 9 | 34 | M | NO | NO | 9 | 6.56 | 1535 | 0 | 7.3 |
| CTR 10 | 56 | F | NO | NO | 29 | 6.78 | 1278 | 0 | 7.4 |
| CTR 11 | 56 | F | NO | NO | 31 | 6.66 | 1400 | 0 | 6.3 |
| CTR 12 | 39 | F | NO | NO | 24 | 6.88 | 1200 | 0 | 7.1 |
| MDD 1 | 48 | F | YES | YES | 24 | 6.36 | 1330 | 6500 | 8.1 |
| MDD 2 | 40 | F | YES | YES | 49 | 6.72 | 1450 | 1000 | 7.5 |
| MDD 3 | 56 | F | NO | NO | 15 | 6.59 | 1370 | 0 | 6.6 |
| MDD 4 | 28 | M | YES | YES | 26 | 6.7 | 1780 | 3000 | 8.4 |
| MDD 5 | 35 | M | YES | YES | 19 | 6.6 | 1335 | 2000 | 7.5 |
| MDD 6 | 32 | F | YES | YES | 19 | 6.7 | 1280 | 100 | 8.4 |
| MDD 7 | 32 | F | YES | NO | 19 | 6.8 | 1470 | 0 | 8.1 |
| MDD 8 | 63 | M | YES | YES | 31 | 6.6 | 1540 | 4000 | 7.9 |
| MDD 9 | 51 | F |  | YES | 36 | 6.3 | 1440 | 700 | 6 |
| MDD 10 | 35 | M | YES | YES | 36 | 6.6 | 1710 | 0 | 7.9 |
| MDD 11 | 44 | M | NO | NO | 24 | 6.52 | 1550 | 3000 | 7.6 |
| MDD 12 | 56 | M | YES | NO | 38 | 6.59 | 1365 | 1000 | 7.5 |
| MDD 13 | 33 | M | YES | NO | 25 | 6.86 | 1640 | 0 | 7.4 |
| MDD 14 | 34 | M | YES | NO | 24 | 6.79 | 1425 | 0 | 8.5 |
| MDD 15 | 45 | F | NO | NO | 29 | 6.9 | 1350 | 0 | 8.5 |
| MDD 16 | 53 | M | NO | NO | 21 | 6.64 | 1520 | 0 | 8.2 |
| MDD 17 | 62 | M | YES | YES | 65 | 6.57 | 1490 | 0 | 7.9 |
| MDD 18 | 36 | F | NO | YES | 32 | 6.74 | 1270 | 2500 | 8 |
| MDD 19 | 40 | M | NO | YES | 52 | 6.48 | 1590 | 3000 | 6.8 |
| MDD 20 | 28 | F | YES | YES | 40 | 6.68 | 1430 |  | 6.7 |
| MDD 21 | 45 | M | YES | NO | 29 | 6.75 | 1514 | 0 | 7.7 |
| MDD 22 | 24 | M | YES | NO | 21 | 6.61 | 1737 |  | 6.9 |
| MDD 23 | 45 | F | YES | NO | 13 | 6.58 | 1170 | 100 | 8.2 |
| MDD 24 | 47 | F | YES | NO | 25 | 6.88 | 1495 | 0 | 6.5 |
| Median | 44.5 |  |  |  | 26.5 | 6.605 | 1460 |  | 7.7 |

Note: ACC: anterior cingulate cortex; BW: brain weight; F: female; M: male; MDD: major depressive disorder; PMI: postmortem interval; RIN: RNA integrity number.

**Table S9b.** Matching of RIN values of ARRAY (left) and Depression (right) SMRI collections

| **Group + area** | **p-value** | **Group + area** | **p-value** |
| --- | --- | --- | --- |
| **BD vs C ACC** | 0.44 | **MDD vs C ACC** | 0.37 |
| **BD vs C DLPFC** | 0.71 | **MDD vs C DLPFC** | 0.36 |
| **BD-P vs C ACC** | 0.33 | **MDD-P vs C ACC** | 0.42 |
| **BD-P vs C DLPFC** | 0.65 | **MDD-P vs C DLPFC** | 0.26 |
| **BD-NP vs C ACC** | 0.82 | **MDD-NP vs C ACC** | 0.49 |
| **BD-NP vs C DLPFC** | 0.90 | **MDD-NP vs C DLPFC** | 0.66 |
| **BD-NP vs BD-P ACC** | 0.54 | **MDD-NP vs MDD-P ACC** | 0.85 |
| **BD-NP vs BD-P DLPFC** | 0.98 | **MDD-NP vs MDD P DLPFC** | 0.70 |
| **BD-S vs C ACC** | 0.96 | **MDD-S vs C ACC** | 0.26 |
| **BD-S vs C DLPFC** | 0.80 | **MDD-S vs C DLPFC** | 0.59 |
| **BD-NS vs C ACC** | 0.25 | **MDD-NS vs C ACC** | 0.54 |
| **BD-NS vs C DLPFC** | 0.74 | **MDD-NS vs C DLPFC** | 0.07 |
| **BD-S vs BD-NS ACC** | 0.22 | **MDD-S vs BD-NS ACC** | >0.9999 |
| **BD-S vs BD-NS DLPFC** | >0.9999 | **MDD-S vs MDD-NS DLPFC** | 0.16 |

Note: ACC; anterior cingulate cortex; BD: bipolar disorder; C: controls; DLPFC, dorsolateral prefrontal cortex; MDD; Major depressive disorder; NP: without psychotic features; NS; patients that did not died of suicide; P: patients with psychotic features: S: patients who completed suicide.

**Table S10**: Primer sequences of PACAP and its receptors

| **Name** | **Sequences** |
| --- | --- |
| PAC1 forward | GGA-GCA-GGACAG-CAA-CCA |
| PAC1 reverse | CCT-CGA-TGA-ACAGCC-AGA-AG |
| VPAC1 forward | TTG-AGG-ATT-ATG-GGT-GCT-GG |
| VPAC1 reverse | AGT-TTC-TGA-AGC-ATT-CGG |
| VPAC2 forward | CGG-CAA-CGA-CCA-GTC-TCA-GT |
| VPAC2 reverse | GAT-GGG-AAA-CAC-GGC-AAA-C |
| CD 38 forward | GAT-GCT-TTC-AAG-GGT-GCA-TTT |
| CD38 reverse | GAA-GAA-TCT-TGT-TGC-AAG-GTA-CG |
| PACAP forward | CTA-GGG-AAG-AGG-TAT-AAA-CAA-AGG-G |
| PACAP reverse | ACG-AGC-GAT-GAC-TGT-TGA-G |

**Table S11a**. Distribution of PACAP Immunoreactivity and in situ hybridization in the human hypothalamus and adjacent areas

| Nucleus | | Marker | PACAP-ir | | | PACAP-mRNA |
| --- | --- | --- | --- | --- | --- | --- |
| *Anterior part of the*  *hypothalamus* | | | Cytoplasmic | Fibre | Basket-  like | Cytoplasmic |
| DBB | Thionine | | +/– | + | + | ++ |
| NBM | Thionine | | +/– | – | – | + |
| LS | Thionine | | +/– | +/– | – | – |
| EA |  | | – | +++ | +++ |  |
| BSTc | SOM | | – | +++ | +++ | – |
| SDN | GAL | | – | +/– | +/– | – |
| Mpoa | GAL | |  | +/– | – | – |
| SCN | AVP | | + | + | – |  |
| PVN | Thionine | | +/– | + | + | +++ |
| Peri | SOM | | ++ | + | – | +++ |
| SON | AVP | | +/– | – | – | +++ |
| *Tuberal region* | | |  |  |  |  |
| VMN | SOM | | – | ++ | ++ | – |
| INF | NPY | | + | + | + | ++ |
| TMN | T | | – | – | – | ++ |
| NTL | SOM | | – | – | – | +/– |
| *Mamillary region* | | |  |  |  |  |
| LMN | T | | + | + |  | +++ |
| MMN | T | | + | + |  | – |

Median intensity of PACAP-immunoreactivity (ir) and mRNA in the human hypothalamus. The category assigned to a given brain region corresponds to the predominant staining according to the following scale: - no staining, +/- weak staining, + staining diffuse and transparent, ++ non-transparent, +++ intense opaque staining. AVP=vasopressin, GAL=galanin, NPY= neuropeptide Y, SOM= somatostatin, BSTc, central nucleus of the bed nucleus of the stria terminalis; DBB, diagonal band of Broca; EA, extended amygdala; INF, infundibular nucleus; LMN, lateral mammillary nucleus; LS, lateral septum; MMN, medial mammillary nucleus; Mpoa, medial preoptic area; NBM, nucleus basalis of Meynert; NTL, lateral tuberal nucleus; Peri, periventricular nucleus; PVN, paraventricular nucleus; SCN, suprachiasmatic nucleus; SDN, sexually dimorphic nucleus; SON, supraoptic nucleus; TMN, tuberomamillary nucleus; VMN, ventromedial nucleus.


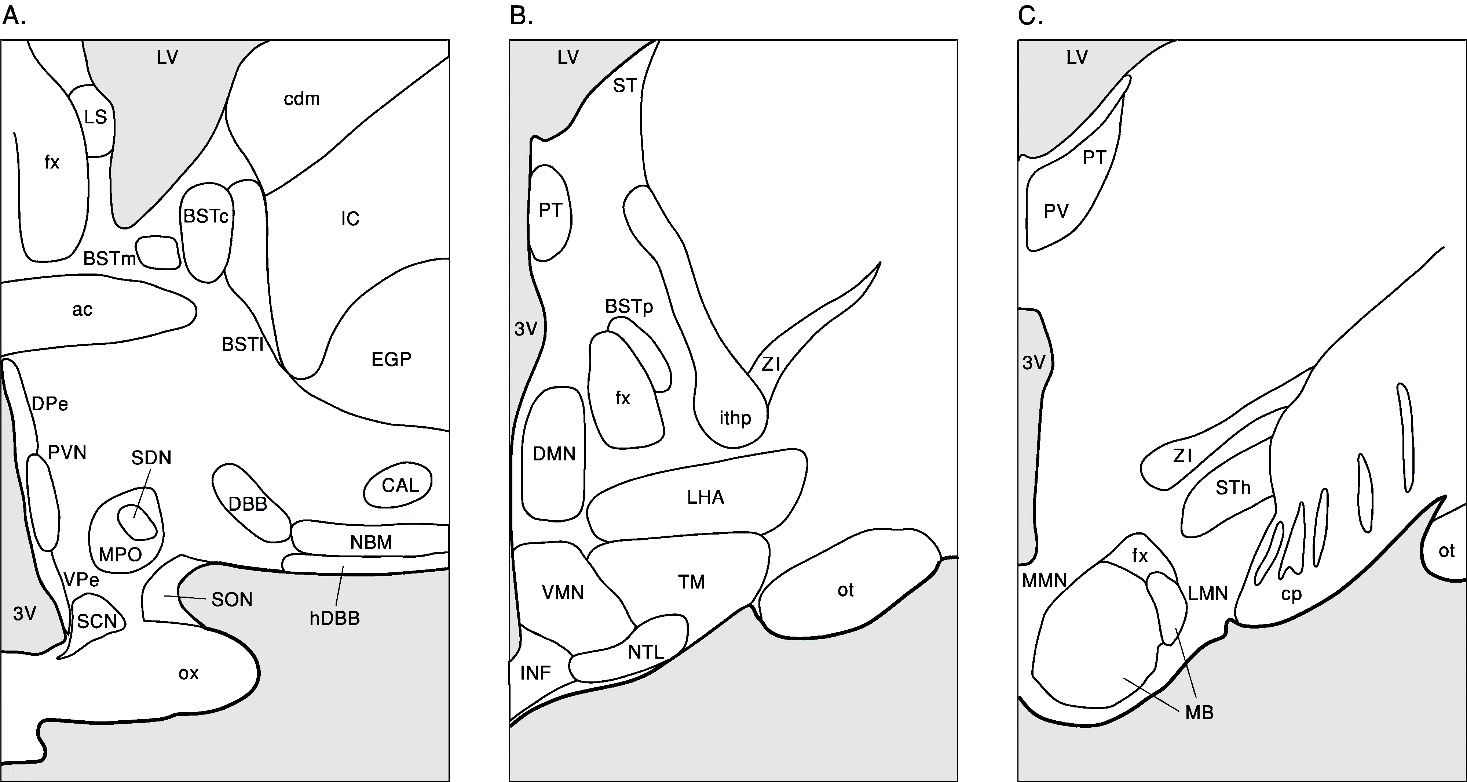
**Figure S11b** (added to table S11). Schematic representation of the nuclei of the human hypothalamus. Abbreviations: Ox: optic chiasma, NBM: nucleus basalis of Meynert, hDBB: horizontal limb of the diagonal band of Broca, SDN: sexually dimorphic nucleus of the preoptic area, SCN: suprachiasmatic nucleus, BST: bed nucleus of the stria terminalis, (c = centralis; m = medialis; l = lateralis; p = posterior); PVN: paraventricular nucleus, SON: supraoptic nucleus, DPe: periventricular nucleus dorsal zone, VPe: periventricular nucleus ventral zone, fx: fornix, 3V: third ventricle, ac: anterior commissure, VMN: ventromedial hypothalamic nucleus, INF: infundibular nucleus, OT: optic tract, MB: mamillary body i.e. MMN: medial mamillary nucleus + LMN: lateromamillary nucleus, cp: cerebral peduncle (Adapted from Swaab, 2003; Fig. 1.6).

**Table S12**: PACAP related gene expression in the DLPFC and ACC comparing female (F) and male (M) controls (Left), female and male MDD patients

|  | Fold change | p-value | BHadj-p | Fold change | p-value | BHadj-p |
| --- | --- | --- | --- | --- | --- | --- |
| **DLPFC target genes** | **Ctr F vs. Ctr. M** | | | **MDD F vs. MDD M** | | |
| PAC1 | -2.53 | 0.24 |  | 1.20 | 1.00 |  |
| VPAC2 | 3.95 | 0.32 |  | -1.60 | 0.57 |  |
| PACAP | -1.61 | 0.80 |  | -1.23 | 0.48 |  |
| VPAC1 | 1.27 | 0.90 |  | -1.74 | 0.36 |  |
| CD38 | -1.79 | 0.73 |  | -2.04 | **0.04** | 0.21 |
| **ACC target genes** |  |  |  |  |  |  |
| PAC1 | 1.19 | 0.94 |  | 1.57 | 0.11 |  |
| VPAC2 | -1.50 | 0.77 |  | 1.72 | 0.14 |  |
| PACAP | -2.03 | 0.95 |  | -1.09 | 0.97 |  |
| VPAC1 | 1.19 | 0.94 |  | 1.57 | 0.11 |  |
| CD38 | -1.40 | 0.53 |  | -1.56 | 0.52 |  |

Note: ACC: anterior cingulate cortex; BHadj-P: P value following Benjamini-Hochberg's adjustment (Benjamini & Hochberg, 1995); Ctr: control; CD38: cyclic adenosine diphosphate (ADP) ribose hydrolase; DLPFC: dorsolateral prefrontal cortex; F, women; M, men; MDD: major depressive disorder; -before fold change = higher expression in women, PAC1, PACAP type I receptor; VPAC1 and VPAC2; vasoactive intestinal peptide (VIP) receptors 1 and 2.

**Table S13**: PACAP related gene expression in the DLPFC and ACC between female (F) and male (M) controls (Ctr) (Left) and female and male BD patients (Right) female and male BD

|  | Fold change | p-value | BHadj-p | Fold change | p-value | BHadj-p |
| --- | --- | --- | --- | --- | --- | --- |
| **DLPFC target genes** | **Ctr F vs. Ctr. M** | |  | **BD F vs. BD M** | | |
| PAC1 | 1.20 | 0.45 |  | -1.13 | 0.21 |  |
| VPAC2 | -1.60 | 0.29 |  | 2.10 | 0.21 |  |
| PACAP | -1.23 | 0.92 |  | 1.44 | 0.15 |  |
| VPAC1 | -1.74 | 0.80 |  | -1.18 | 0.45 |  |
| CD38 | -2.04 | 0.65 |  | -1.30 | 0.87 |  |
| **ACC target genes** |  |  |  |  |  |  |
| PAC1 | 1.01 | 0.89 |  | -1.52 | 0.07 |  |
| VPAC2 | -1.01 | 0.65 |  | -1.40 | **0.03** | 0.07 |
| PACAP | -1.16 | 0.92 |  | -2.05 | **0.03** | 0.07 |
| VPAC1 | 1.42 | **0.03** | 0.15 | -1.28 | 0.053 |  |
| CD38 | -1.22 | 0.42 |  | -2.20 | 0.06 |  |

Note: ACC: anterior cingulate cortex; BHadj-P: P value following Benjamini-Hochberg's adjustment (Benjamini & Hochberg, 1995); CD38: cyclic adenosine diphosphate (ADP) ribose hydrolase; DLPFC: dorsolateral prefrontal cortex; BD: bipolar disorder; - before fold change, = higher expression in women; PAC1, PACAP type I receptor; VPAC1 and VPAC2; vasoactive intestinal peptide (VIP) receptors I and II.

**Table S14**: Correlation analysis between pH and gene expression

|  | CTR | BD | BD-S | BD-N | BD-P | BD-NP |  | CTR | MDD | MDD-S | MDD-N | MDD-P | MDD-NP |
| --- | --- | --- | --- | --- | --- | --- | --- | --- | --- | --- | --- | --- | --- |
| ACC genes |  |  |  |  |  |  | ACC genes |  |  |  |  |  |  |
| CD38 | 0.20 | 0.83 | 0.88 | **- 0.03** | 0.30 | 0.23 | CD38 | 0.23 | 0.97 | 0.49 | 0.50 | 0.13 | 0.79 |
| PACAP | 0.64 | +**0.003** | 0.27 | +**0.03** | +**0.007** | 0.07 | PACAP | 0.37 | 0.51 | 0.46 | 0.10 | 0.11 | 0.58 |
| VPAC1 | 0.40 | +**0.03** | 0.58 | 0.48 | 0.95 | 0.34 | VPAC1 | 0.30 | 0.15 | 0.46 | 0.42 | 0.35 | 0.28 |
| PAC1 | 0.92 | 0.28 | 0.81 | **- 0.03** | 0.06 | 0.12 | PAC1 | 0.37 | 0.51 | 0.46 | 0.10 | 0.35 | 0.28 |
| VPAC2 | 0.40 | +**0.02** | 0.91 | 0.29 | 0.29 | 0.56 | VPAC2 | 0.48 | 0.72 | 0.96 | 0.71 | 0.69 | 0.24 |
| DLPFC genes | |  |  |  |  |  | DLPFC genes | |  |  |  |  |  |
| CD38 | 0.10 | 0.99 | 0.68 | 0.99 | 0.44 | 0.99 | CD38 | 0.46 | 0.14 | 0.24 | 0.71 | 0.36 | 0.19 |
| PACAP | 0.27 | 0.25 | 0.20 | 0.25 | 0.66 | 0.15 | PACAP | 0.34 | 0.58 | 0.19 | 0.30 | 0.61 | 0.94 |
| VPAC1 | 0.22 | 0.81 | 0.32 | 0.81 | 0.32 | 0.99 | VPAC1 | 0.30 | 0.27 | 0.21 | 0.50 | 0.66 | 0.63 |
| PAC1 | 0.87 | 0.72 | 0.30 | 0.72 | 0.17 | 0.098 | PAC1 | 0.32 | 0.41 | 0.26 | 0.56 | 0.33 | 0.97 |
| VPAC2 | 0.39 | 0.82 | 0.27 | 0.82 | 0.58 | 0.92 | VPAC2 | 0.82 | 0.32 | 0.11 | 0.50 | 0.43 | 0.83 |

Note: Values represent p values. ACC: anterior cingulate cortex; BD: bipolar disorder; Ctr: control; DLPFC: dorsolateral prefrontal cortex; F: female; MDD: major depressive disorder; M: male; N: died of natural causes; NP: without psychotic features; P: psychotic features; PMD: post-mortem delay; S: suicide completers; Red/bold: significant correlations, +: positive correlation; -: negative correlation.

**Table S15**: Correlation analysis between fluphenazine doses (in milligrams) and gene expression

|  | BD | BD-S | BD-N | BD-P | BD-NP |  | MDD | MDD-S | MDD-N | MDD-P | MDD-NP |
| --- | --- | --- | --- | --- | --- | --- | --- | --- | --- | --- | --- |
| ACC genes |  |  |  |  |  | ACC genes |  |  |  |  |  |
| CD38 | 0.29 | 0.45 | 0.22 | 0.56 | +**0.01** | CD38 | 0.26 | 0.7 | 0.37 | 0.11 | 0.8 |
| PACAP | 0.33 | 0.84 | 0.09 | 0.34 | 0.16 | PACAP | 0.42 | 0.84 | 0.1 | 0.17 | 0.19 |
| VPAC1 | 0.6 | 0.1 | 0.47 | 0.11 | 0.9 | VPAC1 | 0.15 | 0.46 | 0.17 | 0.11 | 0.38 |
| PAC1 | 0.6 | 0.4 | 0.09 | 0.75 | 0.31 | PAC1 | 0.42 | 0.84 | 0.1 | 0.11 | 0.38 |
| VPAC2 | 0.97 | 0.13 | 0.64 | 0.79 | 0.64 | VPAC2 | 0.32 | 0.31 | 0.9 | **-0.008** | 0.22 |
| DLPFC genes | |  |  |  |  | DLPFC genes | |  |  |  |  |
| CD38 | 0.49 | 0.9 | 0.66 | 0.22 | 0.48 | CD38 | 0.97 | 0.4 | 0.59 | 0.2 | 0.62 |
| PACAP | 0.21 | 0.47 | 0.33 | 0.99 | 0.56 | PACAP | 0.33 | 0.43 | 0.75 | 0.25 | 0.56 |
| VPAC1 | 0.36 | 0.78 | 0.38 | 0.84 | 0.78 | VPAC1 | 0.44 | 0.07 | 0.17 | **-0.006** | 0.73 |
| PAC1 | 0.1 | 0.61 | 0.29 | 0.85 | 0.56 | PAC1 | 0.45 | 0.2 | 0.34 | 0.8 | 0.19 |
| VPAC2 | **-0.04** | 0.75 | **-0.02** | **-0.01** | 0.3 | VPAC2 | 0.5 | 0.13 | 0.15 | **-0.047** | 0.93 |

Note: Values represent P values. ACC: anterior cingulate cortex; BD: bipolar disorder; Ctr: control; DLPFC: dorsolateral prefrontal cortex; F: female; MDD: major depressive disorder; M: male; N: died of natural causes; NP: without psychotic features; P: psychotic features; PMD: post-mortem delay; S: suicide completers; Red/bold: significant correlations; +: positive correlation; -: negative correlation.

**Supplementary (S) Materials and Methods (M)**

**Supplementary (S) Materials and Methods (M)**

**PATIENTS**

**SM1: Alzheimer patients**

Patients with major neuropathological co-morbidity were excluded, while 23 demented patients fulfilled the criteria of a clinical diagnosis of probable Alzheimer’s disease of NINCDS-ADRDA (McKhann et al., 1984) and the neuropathological criteria of CERAD (Mirra et al., 1991) for AD. Post-mortem evaluation took place to establish the distribution of AD changes according to Braak & Braak (1991). The presence of depressive symptoms in AD was established by the Cornell scale for depression in dementia, and the presence of depressive symptoms was established by DSM-IIIR criteria (Alexopoulos et al., 1988).

**SM2: Exclusion criteria for all SMRI specimens**

Exclusion criteria for all SMRI specimens included “significant structural brain pathology on post-mortem examination by a qualified neuropathologist or by pre-mortem imaging; history of significant focal neurological signs; pre-mortem history of a disease of the central nervous system that could be expected to alter gene expression in a persistent way; documented IQ < 70; poor RNA quality. Additional exclusion criteria for unaffected controls included: age less than 30 (thus, still in the period of maximum risk of depression); substance abuse within 1 year before death or significant alcohol-related changes in the liver” (*The Stanley Medical Research Institute, Brain Research Tissue Repository, Array Collection* , 2019).

**Protocols**

**SM3: Immunocytochemical staining protocol**

After deparaffinization in xylene and rehydration in graded ethanols, the sections were rinsed in distilled water. The sections were then heated in the microwave in 0.05 M Tris-HCl pH 9 at full power (700 W) for 10 minutes for antigen retrieval. They were cooled down on a rocking table for 30-60 minutes and rinsed a few times in TBS (0.05 M Tris-HCl pH 7.6, 0.15 M NaCl). Next, a polyclonal rabbit-anti-PACAP antibody (Lot. A14722, Cat. No. T-4473, 1-38, Peninsula Laboratories Inc., RRID: AB_519166) was applied at a dilution of 1:1500 in supermix (SUMI: TBS containing 0.5% Triton X-100 and 0.25% gelatine) with 3% milk powder. Incubation was begun for one hour at room temperature and continued overnight at 4°C. After incubation with the primary antibody, the sections were washed with TBS, followed by incubation for 60 minutes at room temperature with a biotinylated horse anti-rabbit IgG secondary antibody (Lot. ZF0314. Cat. No. BA 1100, Vector Laboratories, RRID: AB_2336201) at 1:400 in SUMI. After washing, the sections were incubated for 60 minutes at room temperature with a 1:800 diluted avidin-biotin complex (ABC) in SUMI. Following washing, an immune signal was developed in a DAB-nickel solution (TBS containing 0.5 mg/ml DAB, 0.01% H2O2, 0.23% ammonium nickel sulphate) for 10 minutes. The reaction was stopped in TBS. After dehydration in graded ethanols and clearing in xylene, the sections were coverslipped with Entellan.

**Photomicrographs**

The photomicrographs of stained sections were taken with the help of the Zeiss Axiovert 200M microscope and with the EXi Aqua Bio-Imaging Microscopy Camera using the Image-Pro Plus software. The images, representing the hypothalamic distribution of PACAP, were collected with x40 objective of the microscope (calibration: 0.77 pixel/ µm).

**SM4: RT-qPCR protocol**

The primer sequences (Table 2) were checked by BLAST for cross reactivity, and were found to be specific for the target genes.

The qPCR experiments were begun by determining the primer efficiencies. This was done by creating a cDNA pool through the collection of 1 μL cDNA from different ACC and DLPFC samples. Subsequently, the pooled cDNA was tested at different dilutions, ranging from 1:1 to 1:64 (in two-fold increments). Of each dilution of pooled cDNA, 1 μL was used in a qPCR reaction, consisting of SYBR Green PCR master mix (Lot. No 1903523, Applied Biosystems, CA, USA) and a mixture of forward and reverse primers (each at a final concentration of 0.15 µM) in a total volume of 10 μL. Each qPCR cycle comprised the following steps: 2 minutes at 50°C; 10 minutes at 95°C; 1 minute at 60°C; 15 seconds at 95°C; 1 minute at 60°C; and 15 seconds at 96°C (Applied Biosystems 7300 RealTime PCR system). The efficiency of the primers was calculated using the inverse logarithm of the dilutions. The slope was calculated, and after that, the efficiency was calculated by efficiency = 1/-slope.

To determine the alterations in the PACAP, PAC1, VPAC1 and VPAC2 and CD38 mRNA expression in the MDD, BD and control patients, each qPCR reaction (10 µl volume, see above) was carried out with cDNA, equivalent to a total RNA input of 5 ng. The data were acquired and processed automatically by the Applied Biosystems 7300 Real Time PCR System.

As negative controls, non-template (NTC) and non-reverse transcriptase (-RT) controls were included. The NTC consisted of replacing the cDNA template with sterile water, while the RT mix was made by omitting reverse transcriptase during the cDNA synthesis. The -RT control serves as a check for contamination with traces of genomic DNA. The chance of influencing the results by variability in the samples was reduced by using measurements of stable reference genes. Six reference genes were used: alpha tubulin (TUBα); tubulin beta-1 chain (TUBβ); ubiquitin-conjugating enzyme (UBC); glyceraldehyde 3-phosphate dehydrogenase (GAPDH); beta Actin (ACTB); hypoxanthine phosphoribosyltransferase 1 (HPRT-1).

**SM5: In situ hybridization protocol**

A 19-mer Locked Nucleic Acid (LNA) probe, consisting of 3 locked bases within a -2-O-methylRNA backbone, was designed using the SiDESIGN tool and targeted against bases 613-595 of the human PACAP mRNA sequence (Accession No. NM_001099733.2). The probe was tagged at the 5’ end with a fluorescein (FAM) group and the sequence was as follows:

FAM 5’ LTmAmG    mCmUmG   LTmCmC    mGmUmG   LAmAmG   mAmUmC  mC 3’. L and m denote locked and methylRNA bases, respectively. The sequence was run through BLAST to check for cross alignments with other transcripts.

A scrambled probe and an irrelevant probe targeting a gene not found in the human genome were used as negative controls. The irrelevant probe was directed against beta lactamase, the gene that confers ampicillin resistance in Lactococcus bacteria. All the probes were custom ordered (Eurogentec, Belgium).

The sections were deparaffinized in xylene, rehydrated in graded ethanols and washed in phosphatase-buffered saline (PBS). The sections were then equilibrated for 2 minutes in 0.01 M sodium citrate buffer pH 6.0, and heated in a microwave until boiling for 2x5 minutes (800 W) to retrieve the mRNA target binding sites. After cooling down, the sections were deproteinated with 0.2N HCl for 20 minutes and incubated for 15 minutes with prewarmed proteinase K at 2 µg/ml at 37°C. The reaction was stopped in glycine. Next, the sections were delipidated in 1x PBS/0.1% Triton X-100 for 10 minutes, followed by a 1-2 hour prehybridization in a hybridization buffer (HBF: 10 mM HEPES buffer pH 7.5, 50% formamide, 600 mM NaCl, 5xDenhardt’s, 1 mM EDTA, 200 µg/ml denatured herring sperm DNA (Invitrogen Life Technologies, Cat. No.15634-017) at 55°C in a humidified chamber covered with Nescofilm. For hybridization, each LNA probe was diluted to 50 nM in HBF, denatured for 10 minutes at 95°C and cooled down on ice. After pouring off the hybridization buffer from the prehybridization step, the diluted probe was applied to the sections. Hybridization was carried out at 55°C overnight in a humidified chamber. The overnight incubation was followed by stringency washes for 5 minutes each, 2x standard sodium citrate buffer (20xSSC: 3M NaCl, 0.3M trisodium citrate dihydrate, pH 7) at 37°C, 2x SSC at 55°C, 0.5x SSC at 55°C, and 0.2 SSC at 55°C. After brief washes in TBS, immunological detection was carried out by incubating the sections with anti-fluorescein-alkaline phosphatase (AP), a conjugated antibody (Roche Life Science, Cat. No.11426338910, RRID: AB_514504) diluted 1:3000 in super mix (SUMI: 0.05 M Tris-HCl pH 7.6, 0.15 M NaCl, 0.25% [w/v] gelatine, 0.5% [v/v] Triton X-100) for 3 hours at RT under dark conditions. The sections were then washed 3x5 minutes in 1x TBS and buffer 2 (100 mM Tris-HCl pH 9.5, 100 mM NaCl, 5 mM MgCl2). ISH signal was developed in NBT/BCIP substrate solution (0.34mg/ml NitroBlueTetrazolium Chloride [NBT; Roche Life Science, Cat. No.1585029001], 0.17 mg/ml 5-bromo-4-chloro-3-indolyl phosphate [BCIP; Roche Life Science Cat. No.11585002001], 0.24 mg/ml levamisole (Sigma-Aldrich, Cat. No. L9756) in buffer 2) under dark conditions at RT overnight. The reactions were stopped in distilled water and the sections were washed with 100% methanol for 3.5 minutes on a rocking table to remove the brown discoloration. Following washes in distilled water, the sections were coverslipped with glycerol.

In situ hybridization procedure was performed following early research of our group (Alkemade, Unmehopa, Wiersinga, Swaab, & Fliers, 2005).

**Photomicrographs**

The photomicrographs were taken with the help of the Zeiss Axiovert 200M microscope and with the EXi Aqua Bio-Imaging Microscopy Camera using the Image-Pro Plus software. The images, representing the hypothalamic distribution of PACAP mRNA, were collected with x40 or x63 oil objective of the microscope (calibration 11.97 pixel/µm).

**SM6: Western blot protocol**

The N-terminal 28 amino acids of PACAP-38 show a considerable sequence homology of 68% with vasoactive intestinal polypeptide (VIP). To demonstrate that the anti-PACAP-38 antibody used for immunocytochemical staining specifically targets human PACAP-38 and does not cross-react with VIP, a Western blot was performed using PACAP-38 (Anaspec Inc., Cat. No. AS-22519) and VIP (Merck, Cat. no. V6130) peptides as positive controls for rabbit polyclonal anti-PACAP-38 (Peninsula Laboratories Inc., Cat. No. T-4473, Lot A18166, RRID: AB_519166) and rabbit anti-VIP (VIPER (bleeding 18-09-1986, raised at the Netherlands Institute for Neuroscience, Amsterdam)) primary antibodies.

Prior to blotting, 10 µl of SeeBlue Plus2 Pre-stained Protein Standard (Thermo Fisher Scientific, Cat. No. LC5925) and 80 ng of each peptide were loaded to Novex 16% Tricine gel (Thermo Fisher Scientific, Cat. No. EC66952BOX) and SDS polyacrylamide gel electrophoresis was run using SDS running buffer (100 mM Tricine, 100 mM Tris base, 0.1% SDS). Electrophoresis was carried out in reducing conditions for 180 minutes at 50-80 V. The proteins were then transferred to 0.2 µm PVDF membrane (Millipore, Cat. No. ISEQ10100) for 90 minutes at 25 V. The membrane was blocked in 5% non-fat dried milk in TBST (20 mM Tris base, 150 mM NaCl, and 0.1% Tween 20) for 1 hour at room temperature. After rinsing in double distilled water, the membrane was probed with the anti-PACAP-38 antibody at a dilution of 1:1500 in TBST with 1% BSA overnight at 4°C. The membrane was washed in TBST and probed with a horseradish peroxidase (HRP)-conjugated goat anti-rabbit secondary antibody (Thermo Fisher Scientific, Cat. No. 32460, RRID: AB_1185567) at 1:5000 dilution in TBST with 5% non-fat dried milk for 1 hour at room temperature. After washes in TBST and double distilled water, chemiluminescent detection was performed using Pierce ECL Western Blotting substrate (Thermo Fisher Scientific, Cat. No. 32106) and visualized with Fusion FX imaging system (Vilber, France) (For results see Figure S1). To assay with anti-VIP primary antibody, the membrane was washed briefly in TBST, then stripped with Restore Western Blot Stripping Buffer (Thermo Fisher Scientific, Cat. No. 21059) for 10 minutes at room temperature and blocked in TBST with 5% non-fat dried milk for 1 hour at room temperature. After rinsing in double distilled water, the membrane was re-probed using the anti-VIP antibody at a dilution of 1:1500 in TBST with 1% BSA at 4°C overnight. Following four washes with TBST, the membrane was incubated with the same HRP-conjugated goat anti-rabbit secondary antibody as previously for 1 hour at room temperature. Chemiluminescence was performed using Pierce ECL Western Blotting substrate and visualized with Fusion FX imaging system. For results see figure S1.

Despite 68% sequence homology between PACAP-38 and VIP, the anti-PACAP-38 antibody clearly reacted only with PACAP-38, and not with VIP peptide.

##
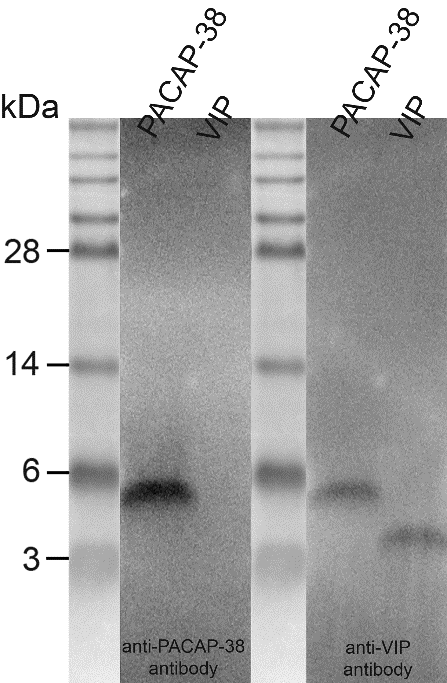
Fig. SM1: Western blot analysis excluding cross-reactivity between the anti-PACAP-38 antibody and VIP peptide.

**SM7. Adsorption of the anti-PACAP antiserum with VIP peptide**

To investigate whether removal of a potential fraction of VIP-cross reacting antibodies within the anti-PACAP antiserum would affect PACAP staining pattern in the central nucleus of the bed nucleus of the stria terminalis (BSTc) (known for its clear staining of both, PACAP and VIP), we performed a solid-phase adsorption of the anti-PACAP antiserum with VIP peptide spotted onto nitrocellulose. The anti-PACAP antibody was diluted to 1:1500 in supermix and incubated consecutively with 3 nitrocellulose strips, each containing 10 µg of VIP peptide. Each incubation was started at room temperature for an hour followed by overnight at 4^0^C. The following day, a proportion of the adsorbed antibody solution was collected and kept at 4^0^ C to monitor removal of the VIP-cross reacting antibodies. The remaining adsorbed antibody solution was used to start the next adsorption cycle. After completion of all cycles, the collected adsorbed antibody solutions were reacted with nitrocellulose strips, spotted with a dilution series of VIP and PACAP peptide These strips were designated as ‘control strips’. The VIP control strip served as a validation for clearance of VIP-cross reacting antibodies, while the PACAP strip was included to determine whether VIP adsorption would alter binding to PACAP. Immunological detection of dots was performed using biotinylated antibodies, ABC and DAB nickel. To investigate the effect of VIP adsorption on the staining pattern in the BSTc, sections from two subjects (NBB no. 98-031, 33 year-old male and 99-044, 88-year-old female; both non-demented controls) were incubated with the absorbed antibody solutions, harvested after each adsorption cycle. The procedure employed for staining these sections was based on the conventional PACAP staining protocol used for hypothalamus sections (for details, see section on immunohistochemical procedure). As a reference, adjacent sections were reacted with non-adsorbed anti-PACAP antibody.


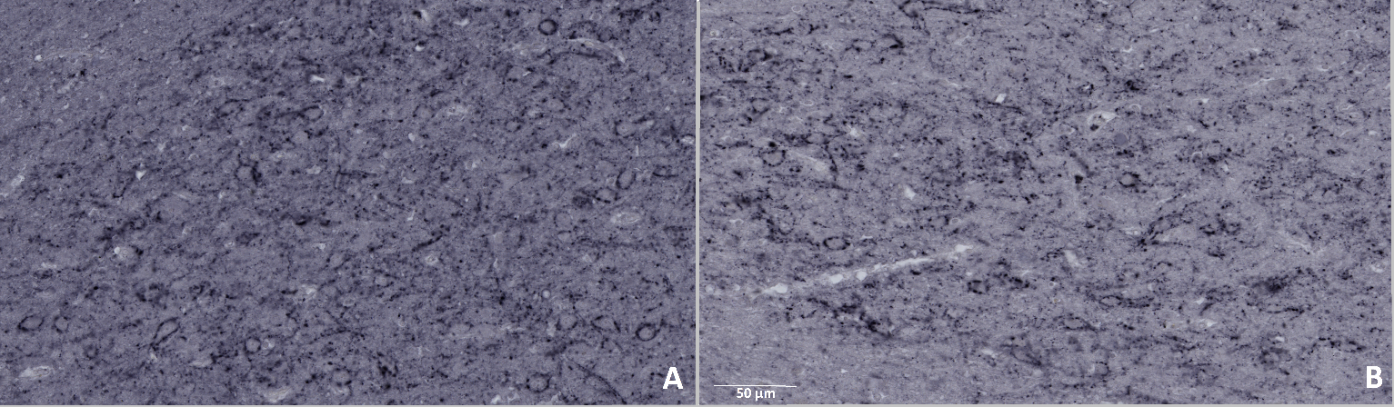


**Figure SM6. Adsorption of the anti-PACAP antiserum to VIP does not affect PACAP immunoreactivity in the BSTc**

Potential VIP cross reacting antibodies were removed from the anti-PACAP antiserum following 3 solid phase adsorptions with VIP. BSTc sections were subsequently stained using adsorbed antibody solution (panel B) and compared to the original, non-adsorbed anti-PACAP antiserum (panel A). The staining pattern after adsorption remains was quite similar to the original staining, indicating that VIP cross reacting antibodies do not add significantly to the immunohistochemical signal produced by the anti-PACAP antiserum. Images shown are taken from subject NBB no. 98-031, 33 year-old male.

**Supplementary (S) Figures Results (R)**


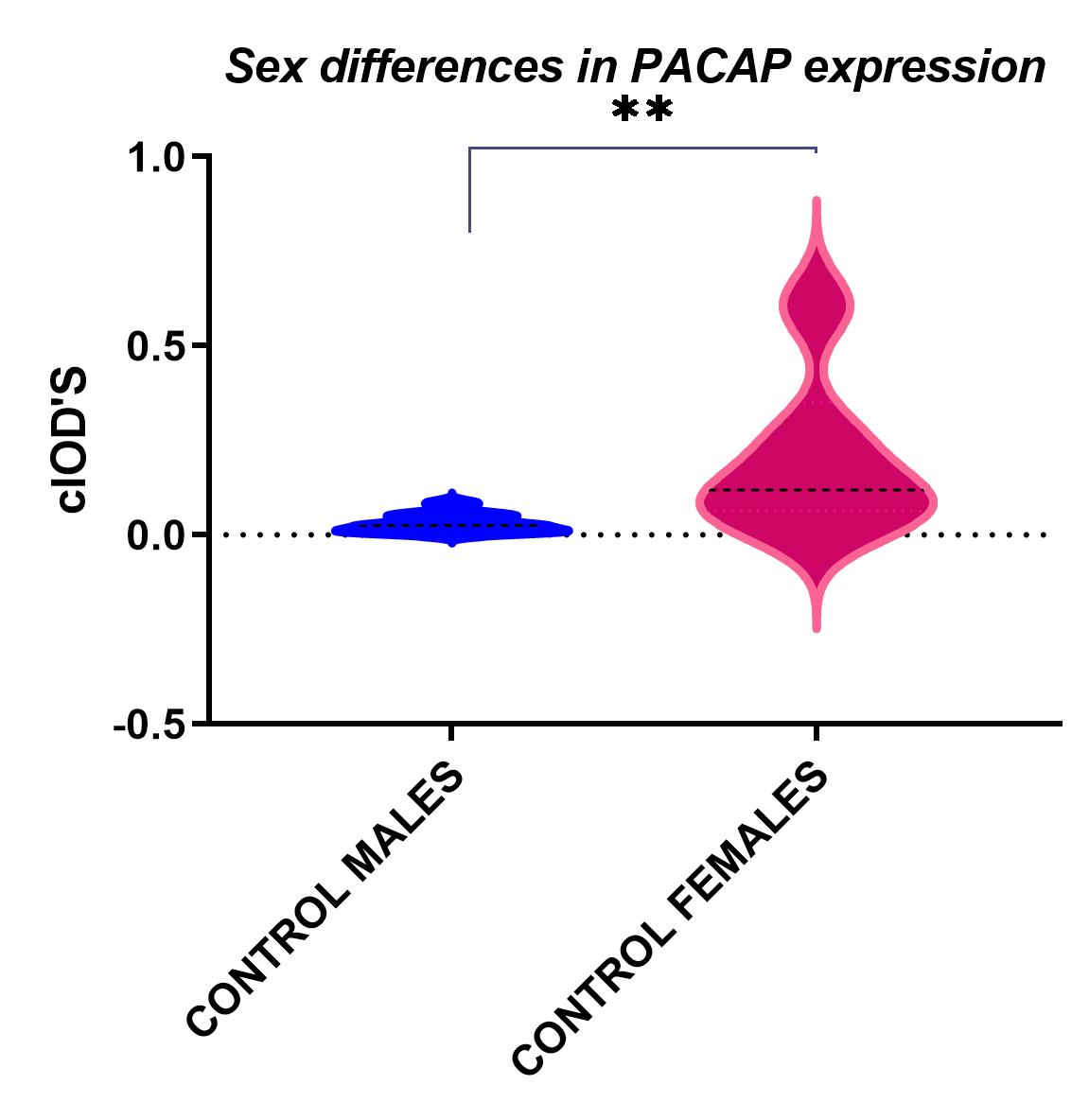

**Fig. SR1**: PACAP-ir expression differences in cIOD (corrected integrated optical density) of male controls (N=11) and female controls (N=6). The data show that women have a significantly higher expression than men (p= 0.0048): **indicates P<0.01.


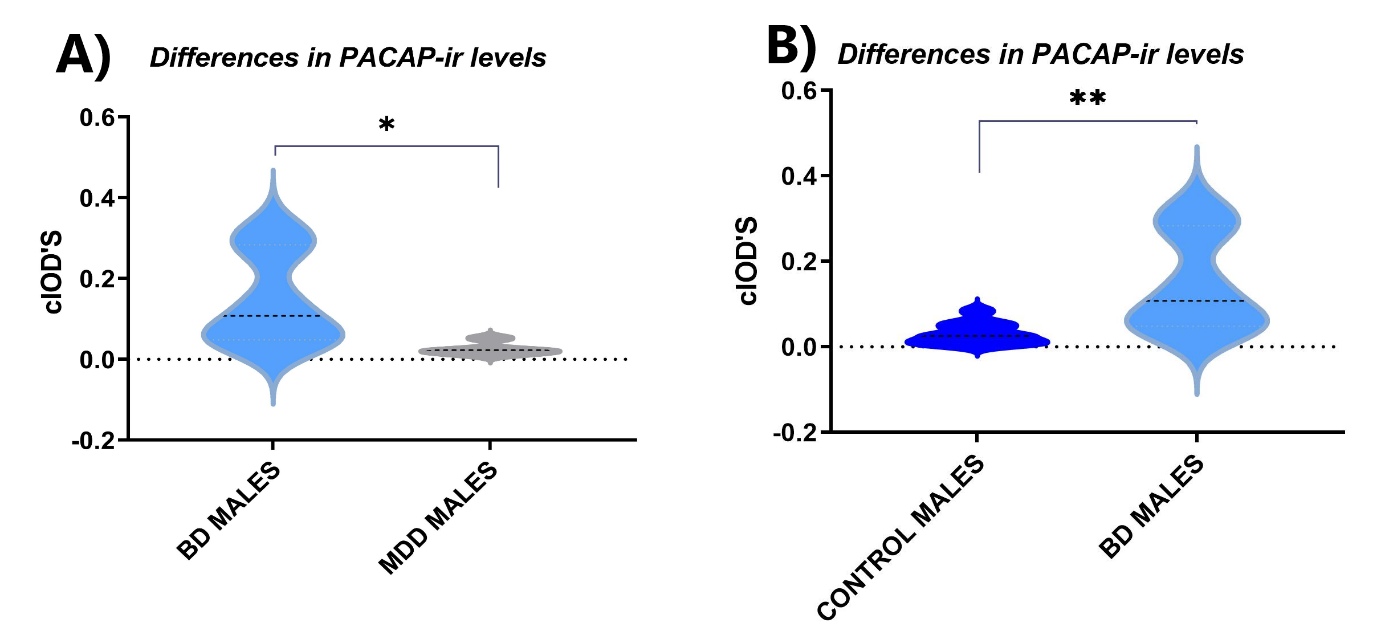


**Fig. SR2:** PACAP-ir expression differences in cIOD. The graphs show a significant difference between bipolar (BD) men (N=5) and major depressive (MDD) men (N=6) (p=0.007) and male controls (N=11) with BD men (N=5) (p=0.017).*indicates P<0.05, ** indicates P<0.01.

**
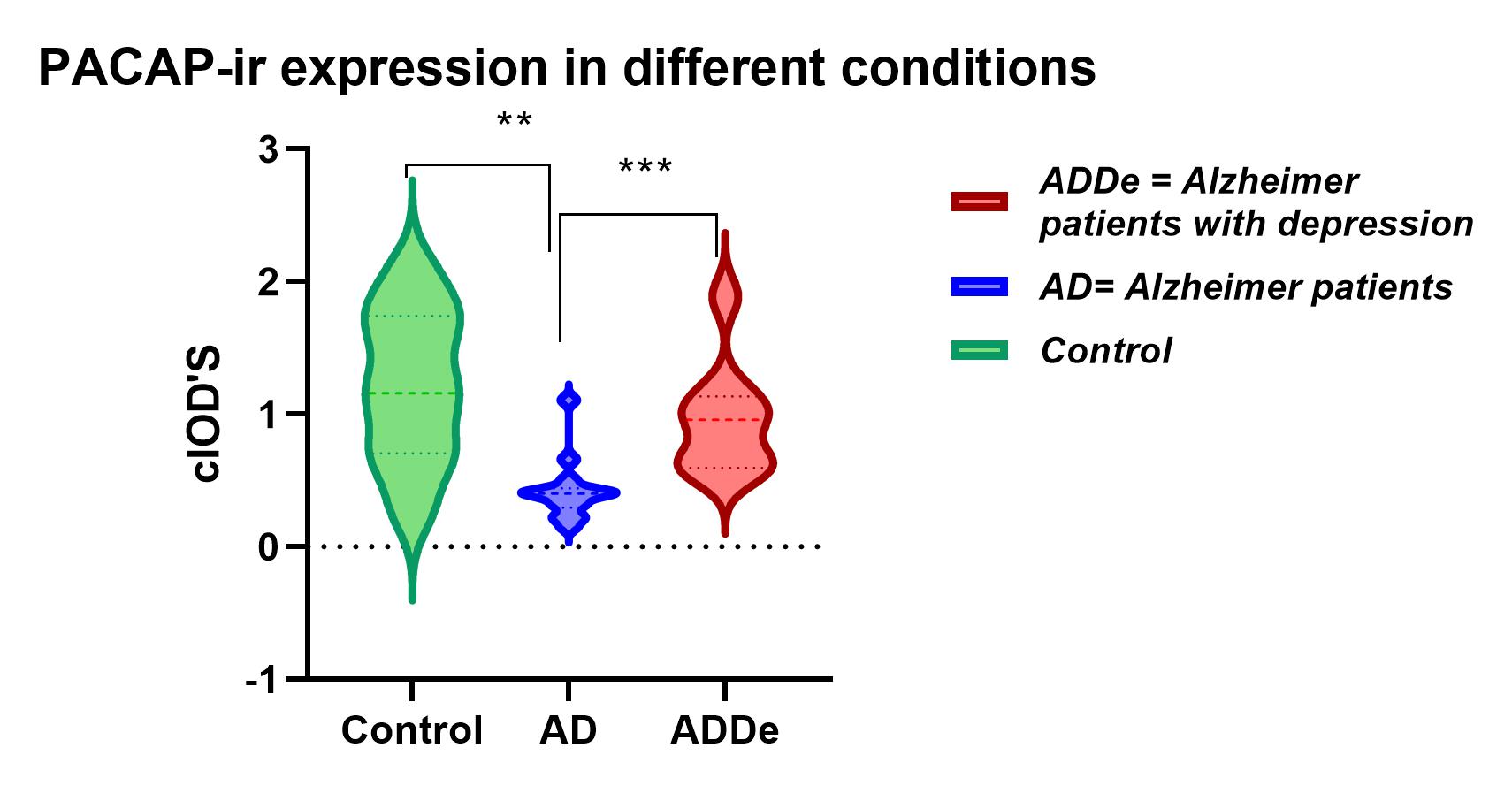
**

**Fig. SR3**: PACAP-IR expression differences in cIOD between the Alzheimer’s patients, Alzheimer’s patients with depression, and the control group. Note that compared to controls, the entire AD group shows a significant reduction in PACAP-ir (p= 0.0012), while AD patients with depression show an increase relative to the entire AD group (p = 0.0003). **indicates P<0.01, ***indicates P<0.001.


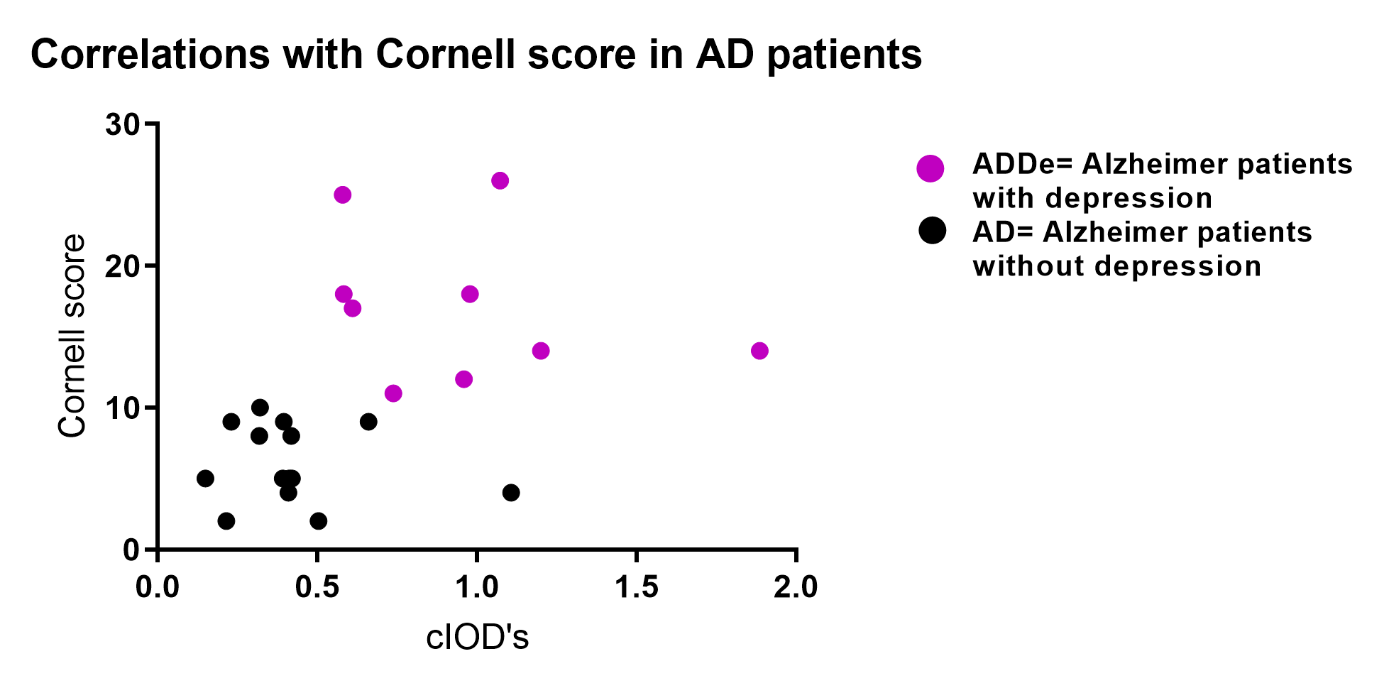


**Figure SR5***:* A positive correlation is present between the Cornell depression score and the expression of PACAP in all AD patients combined (rho= 0.44; p=0.034).

**Supplementary(S) results(R) Figure SR5**

**Fig. SR5.a** Confidence intervals of the comparisons between the gene expression in the dorsolateral prefrontal cortex of bipolar disorder (BD) patients and controls. The Mann-Whitney test was applied to the 10log-transformed RNA concentration data, and the confidence intervals were adjusted according to the Benjamin-Hochberg criterion (Benjamini & Hochberg, 1995). The solid green line denotes zero change. The dashed green lines denote the + or – 1.5 fold changes. The dashed orange lines denote the + or - 2 fold changes. The median values were calculated using the Hodges-Lehmann delta (HLdelta) procedure. This figure corresponds to the data presented in Table A, DLPFC (upper left).

**Fig. SR5.b** Confidence intervals of the comparisons between the gene expression in the anterior cingulate cortex of bipolar disorder (BD) patients and controls. The Mann-Whitney test was applied to the 10log-transformed RNA concentration data, and the confidence intervals were adjusted according to the Benjamin-Hochberg criterion (Benjamini & Hochberg, 1995). The solid green line denotes zero change. The dashed green lines denote the + or – 1.5 fold changes. The dashed orange lines denote the + or - 2 fold changes. The median values were calculated using the Hodges-Lehmann delta (HLdelta) procedure. This figure corresponds to the data presented in Table A, ACC (upper right).

**Fig. SR5.c** Confidence intervals of the comparisons between the gene expression in the dorsolateral prefrontal cortex of major depressive disorder (MDD) patients and controls. The Mann-Whitney test was applied to the 10log-transformed RNA concentration data, and the confidence intervals were adjusted according to the Benjamin-Hochberg criterion (Benjamini & Hochberg, 1995). The solid green line denotes zero change. The dashed green lines denote the + or – 1.5 fold changes. The dashed orange lines denote the + or - 2 fold changes. The median values were calculated using the Hodges-Lehmann delta (HLdelta) procedure. This figure corresponds to the data presented in Table A, DLPFC (lower left).

**Fig. SR6.a** Confidence intervals of the comparisons between the gene expression in the anterior cingulate cortex of major depressive disorder (MDD) patients with (S) and without (NS) completed suicide and controls. The Kruskal-Wallis test was applied to the 10log-transformed RNA concentration data. The global p-values allowed multiple comparisons and construction of confidence intervals for only PACAP. The confidence intervals and corresponding p-values were calculated using the difference of mean ranks (Conover, 1980) and are completely consistent. The solid green line denotes zero change. This figure corresponds to the data presented in Table B2, ACC.

**Fig. SR6.b** Confidence intervals of the comparisons between the gene expression in the dorsolateral prefrontal cortex of bipolar disorder (BD) patients with (S) and without (NS) completed suicide. The Kruskal-Wallis test was applied to the 10log-transformed RNA concentration data. The global p-values allowed multiple comparisons and construction of confidence intervals for only VPAC1. The confidence intervals and corresponding p-values were calculated using the difference of mean ranks (Conover, 1980) and are completely consistent. The solid green line denotes zero change. This figure corresponds to the data presented in Table B3, DLPFC.

**Fig. SR6.c** Confidence intervals of the comparisons between the gene expression in the anterior cingulate cortex of bipolar disorder (BD) patients with (S) and without (NS) completed suicide and controls. The Kruskal-Wallis test was applied to the 10log-transformed RNA concentration data. The global p-values allowed multiple comparisons for PACAP, VPAC1, VPAC2. This multiple testing situation was corrected with the Benjamini-Hochberg false coverage procedure (FCR) (Benjamini & Hochberg, 1995). The confidence intervals and corresponding p-values were calculated using the difference of mean ranks (Conover, 1980) and are completely consistent. The solid green line denotes zero change. The dashed green lines denote the + or – 1.5 fold changes. The dashed orange lines denote the + or - 2 fold changes. This figure corresponds to the data presented in Table B4, ACC

**Fig. SR7.a** Confidence intervals of the comparisons between the gene expression in the anterior cingulate cortex of major depressive disorder (MDD) patients, with (P) and without (NP) psychotic features and controls in this study. The Kruskal-Wallis test was applied to the 10log-transformed RNA concentration data. The global p-values allowed multiple comparisons and construction of confidence intervals for only PACAP. The confidence intervals and corresponding p-values were calculated using the difference of mean ranks (Conover, 1980) and are completely consistent. The solid green line denotes zero change. This figure corresponds to the data presented in Table C1, DLPFC.

**Fig. SR7.b** Confidence intervals of the comparisons between the gene expression in the anterior cingulate cortex of bipolar disorder (BD) patients, with (P) and without (NP) psychotic features and controls. The Kruskal-Wallis test was applied to the 10log-transformed RNA concentration data. The global p-values allowed multiple comparisons and construction of confidence intervals for only PACAP. The confidence intervals and corresponding p-values were calculated using the difference of mean ranks (Conover, 1980) and are completely consistent. The solid green line denotes zero change. This figure corresponds to the data presented in Table C3, DLPFC.

**Fig. SR7.c** Confidence intervals of the comparisons between the gene expression in the anterior cingulate cortex of bipolar disorder (BD) patients, with (P) and without (P) psychotic features and controls. The Kruskal-Wallis test was applied to the 10log-transformed RNA concentration data. The global p-values allowed multiple comparisons and construction of confidence intervals for VPAC1 and VPAC2. This multiple testing situation was corrected with the Benjamini-Hochberg false coverage procedure (FCR) (Benjamini & Hochberg, 1995). The confidence intervals and corresponding p-values were calculated using the difference of mean ranks (Conover, 1980) and are completely consistent. The solid green line denotes zero change. This figure corresponds to the data presented in Table C4, ACC.

**References:**

Alexopoulos, G. S., Abrams, R. C., Young, R. C., & Shamoian, C. A. (1988). Cornell scale for depression in dementia. *Biological Psychiatry*, *23*(3), 271–284. https://doi.org/10.1016/0006-3223(88)90038-8

Alkemade, A., Unmehopa, U. A., Wiersinga, W. M., Swaab, D. F., & Fliers, E. (2005). Glucocorticoids decrease thyrotropin-releasing hormone messenger ribonucleic acid expression in the paraventricular nucleus of the human hypothalamus. *The Journal of Clinical Endocrinology and Metabolism*, *90*(1), 323–327. https://doi.org/10.1210/JC.2004-1430

Benjamini, Y., & Hochberg, Y. (1995). Controlling the False Discovery Rate: A Practical and Powerful Approach to Multiple Testing. *Journal of the Royal Statistical Society: Series B (Methodological)*, *57*(1), 289–300. https://doi.org/10.1111/j.2517-6161.1995.tb02031.x

Braak, H., & Braak, E. (1991). Neuropathological stageing of Alzheimer-related changes. *Acta Neuropathologica*, *82*(4), 239–259. https://doi.org/10.1007/BF00308809

Conover, W. (1980). *Practical nonparametric statistics* (2.). New York: John Wiley & Sons, Inc.

Hoogendijk, W. J. G., Sommer, I. E. C., Pool, C. W., Kamphorst, W., Hofman, M. A., Eikelenboom, P., & Swaab, D. F. (1999). Lack of Association Between Depression and Loss of Neurons in the Locus Coeruleus in Alzheimer Disease. *Archives of General Psychiatry*, *56*(1), 45. https://doi.org/10.1001/archpsyc.56.1.45

McKhann, G., Drachman, D., Folstein, M., Katzman, R., Price, D., & Stadlan, E. M. (1984). Clinical diagnosis of Alzheimer’s disease: Report of the NINCDS-ADRDA Work Group* under the auspices of Department of Health and Human Services Task Force on Alzheimer’s Disease. *Neurology*, *34*(7), 939–939. https://doi.org/10.1212/WNL.34.7.939

Meynen, G., Unmehopa, U. A., Hofman, M. A., Swaab, D. F., & Hoogendijk, W. J. G. (2007). Relation between Corticotropin-Releasing Hormone Neuron Number in the Hypothalamic Paraventricular Nucleus and Depressive State in Alzheimer’s Disease. *Neuroendocrinology*, *85*(1), 37–44. https://doi.org/10.1159/000100582

Mirra, S. S., Heyman, A., McKeel, D., Sumi, S. M., Crain, B. J., Brownlee, L. M., … Berg, L. (1991). The Consortium to Establish a Registry for Alzheimer’s Disease (CERAD): Part II. Standardization of the neuropathologic assessment of Alzheimer’s disease. *Neurology*, *41*(4), 479–479. https://doi.org/10.1212/WNL.41.4.479

Swaab, D. F. (2003). The Human Hypothalamus. Basic and Clinical Aspects. In *Handbook of clinical neurology*. Amsterdam: Elsevier Ltd.

*The Stanley Medical Research Institute, Brain Research Tissue Repository, Array Collection* . (2019). Rockville.
